# PlasmidHostFinder: Prediction of Plasmid Hosts Using Random Forest

**DOI:** 10.1128/msystems.01180-21

**Published:** 2022-04-06

**Authors:** Derya Aytan-Aktug, Philip T. L. C. Clausen, Judit Szarvas, Patrick Munk, Saria Otani, Marcus Nguyen, James J. Davis, Ole Lund, Frank M. Aarestrup

**Affiliations:** a National Food Institute, Technical University of Denmark, Kgs. Lyngby, Denmark; b Consortium for Advanced Science and Engineering, University of Chicago, Chicago, Illinois, USA; c Data Science and Learning Division, Argonne National Laboratory, Argonne, Illinois, USA; d Northwestern Argonne Institute for Science and Engineering, Evanston, Illinois, USA; UCSF

**Keywords:** antimicrobial resistance, horizontal gene transfer, machine learning, plasmid, plasmid host, plasmid host range, random forest

## Abstract

Plasmids play a major role facilitating the spread of antimicrobial resistance between bacteria. Understanding the host range and dissemination trajectories of plasmids is critical for surveillance and prevention of antimicrobial resistance. Identification of plasmid host ranges could be improved using automated pattern detection methods compared to homology-based methods due to the diversity and genetic plasticity of plasmids. In this study, we developed a method for predicting the host range of plasmids using machine learning—specifically, random forests. We trained the models with 8,519 plasmids from 359 different bacterial species per taxonomic level; the models achieved Matthews correlation coefficients of 0.662 and 0.867 at the species and order levels, respectively. Our results suggest that despite the diverse nature and genetic plasticity of plasmids, our random forest model can accurately distinguish between plasmid hosts. This tool is available online through the Center for Genomic Epidemiology (https://cge.cbs.dtu.dk/services/PlasmidHostFinder/).

**IMPORTANCE** Antimicrobial resistance is a global health threat to humans and animals, causing high mortality and morbidity while effectively ending decades of success in fighting against bacterial infections. Plasmids confer extra genetic capabilities to the host organisms through accessory genes that can encode antimicrobial resistance and virulence. In addition to lateral inheritance, plasmids can be transferred horizontally between bacterial taxa. Therefore, detection of the host range of plasmids is crucial for understanding and predicting the dissemination trajectories of extrachromosomal genes and bacterial evolution as well as taking effective countermeasures against antimicrobial resistance.

## INTRODUCTION

Plasmids are extrachromosomal DNA sequences that have crucial roles in bacterial ecology, evolution, and the spread of antimicrobial resistance (AMR) (1). They are typically circular, self-replicating, and transferable and tend to obtain, lose, or rearrange their genetic content rapidly, which make them extremely mosaic, diverse, and plastic. Plasmids are generally composed of backbone and accessory genes. The backbone includes replication (*rep*) and mobility (*mob*) genes, which are relatively conserved among the plasmids of the same family (2). These features have also been used to type and compare plasmids that are isolated from different hosts by using replicon and MOB typing (3–5). The accessory genes generally confer selective advantages to the host, such as AMR, virulence, and metal resistance, increasing host survival under stress conditions despite the metabolic costs that plasmids cause to the host (6). Plasmids also harbor toxin-antitoxin systems and act as parasitic entities (7). Plasmids are often competent horizontal gene transfer vectors and are able to move from one bacterium to another via conjugation, transduction, or transformation, causing persistent genetic exchange between bacterial hosts (1, 8).

Plasmids vary in the number and range of taxa they can transfer to, replicate in, and be maintained in. They can be roughly categorized as having narrow or broad host ranges (9). The features that determine the host range capacity of plasmids are not fully understood yet, but origin of replication, replication initiation dependencies, and origin of transfer are known to be important for host range (9).

Plasmid host ranges can be determined empirically by testing potential hosts *in vitro* (10, 11). However, sequence-based approaches can be used for plasmid host range prediction, which is more practical than the empirical methods in terms of turnaround times and usage of laboratory resources (11). Previous studies have attempted to predict plasmid host ranges by comparing oligonucleotide compositions of plasmids and chromosomes (1, 9, 10, 12–14). Narrow-host-range plasmids are expected to have similar oligonucleotide compositions to the host organism due to plasmid sequence amelioration: e.g., adaptation to a preferred host codon usage (12). However, this method falls short when predicting broad-host-range plasmids because plasmids can often transfer to distantly related hosts (9, 12).

Previously developed plasmid identification tools such as PlasmidFinder and PlasFlow have been developed to determine plasmid hosts (3, 15). PlasmidFinder identifies plasmids in whole-genome sequences by searching against plasmid replicon sequences from the *Enterobacteriaceae* and Gram-positive species. This alignment-based tool identifies plasmids from these taxa with high accuracy by indicating a source organism based on the best-matching replicon. PlasFlow was developed using a deep neural network and trained by the *k*-mer counts of fragments at least 1,000 nucleotides in length, and it can detect plasmid hosts at the phylum level. To our knowledge, the PlasFlow tool is not currently maintained. Recently, in 2021, Redondo-Salvo et al. (16) developed an automated plasmid classification tool called COPLA that works by assigning plasmid taxonomic units (PTUs) based on total average nucleotide identity.

Machine learning, a form of artificial intelligence, has been utilized in recent years to understand various biological systems by detecting the linear and nonlinear correlations between input and output data (17). It has been used to predict phenotypes and structures in nature, and it has the potential to discover unknown features, such as novel AMR genes (18–20). In this study, in order to better predict plasmid hosts and infer plasmid host ranges, we developed a set of random forest-based machine learning models for predicting plasmid hosts at several bacterial taxonomic levels.

## RESULTS

### Plasmid host prediction performances for the PATRIC hold-out data set.

**FIG 1:**
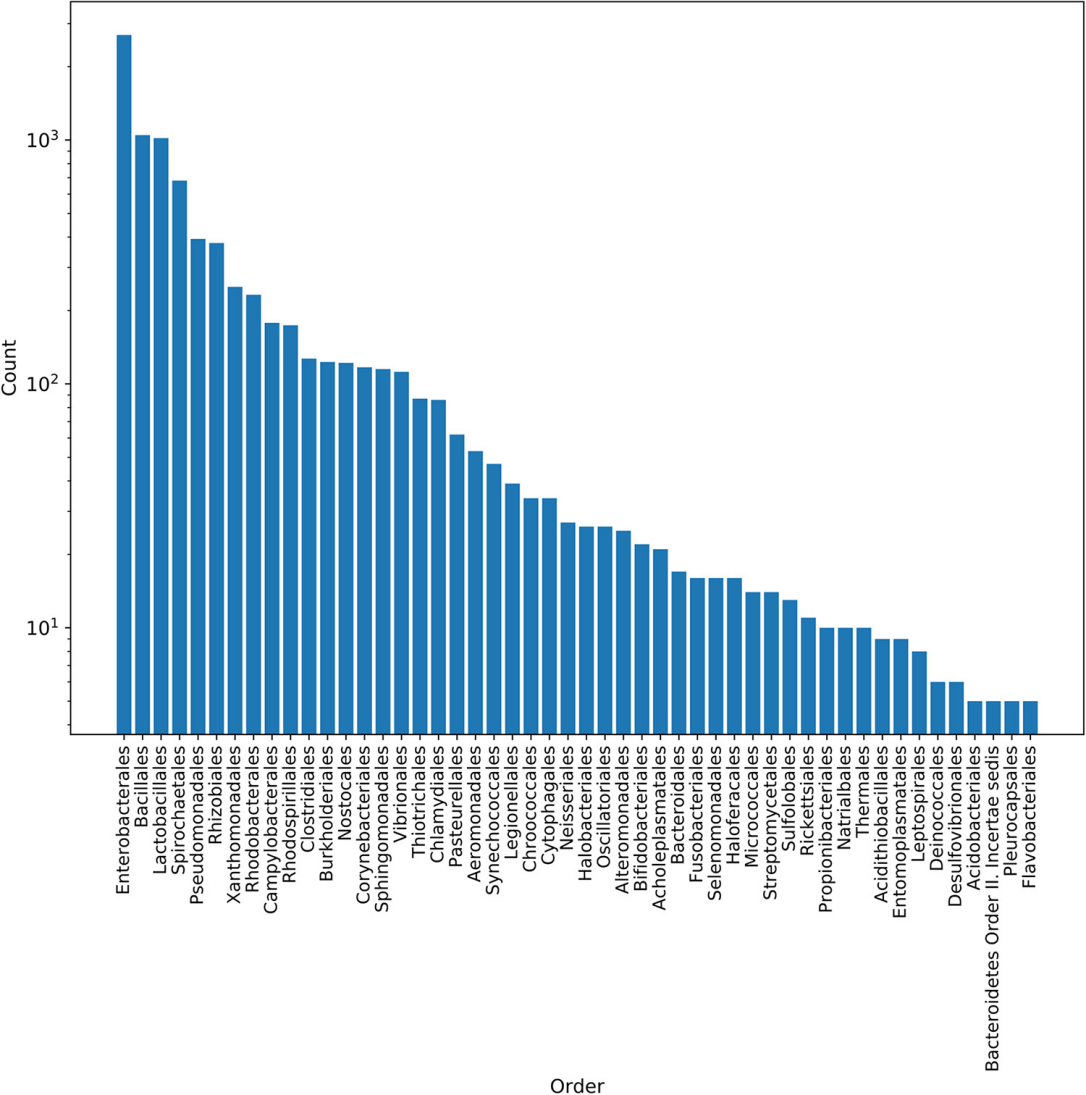
Plasmid host distribution at the order level in the PATRIC data set. The PATRIC plasmid collection was dominated by the *Enterobacterales*, *Bacillales*, and *Lactobacillales* orders, which make up 55.6% of the plasmid hosts.

In order to develop models for predicting the host organisms of plasmids, a total of 8,519 plasmids with at least species-level host information were downloaded and curated from the the Pathosystems Resource Integration Center (PATRIC) database and included in this study (see Table S1B in the supplemental material). These plasmids originate from 359 species belonging to 174 genera, 93 families, and 50 orders (see Fig. S1 and Table S1C in the supplemental material). Most of the plasmids in the collection come from the orders *Enterobacterales*, *Bacillales*, and *Lactobacillales*, which comprise 55.6% of the hosts in the data set (Fig. 1).

10.1128/mSystems.01180-21.1 FIG S1 Neighbor-joining tree. The distance tree was generated using the *k*-mer Hamming distances. The surrounding rings are color coded based on the plasmid hosts. Each ring represents host range at a different taxonomic level, and each color represents a different host. From the inner ring to the outer ring, the taxonomic levels are species, genus, family, and order. If a plasmid is hosted by multiple species, only one of them represented in the tree. All the information regarding the plasmid hosts is available in Table S1B. Download FIG S1, PDF file, 1.5 MB. Copyright © 2022 Aytan-Aktug et al. 2022 Aytan-Aktug et al. https://creativecommons.org/licenses/by/4.0/ This content is distributed under the terms of the Creative Commons Attribution 4.0 International license.

10.1128/mSystems.01180-21.6 Table S1 (A) Random grid search for random forest parameter tuning. The model was tuned for the 8-mers and genus level. (B) PATRIC plasmid IDs and host names at the species level. (C) The PATRIC plasmid hosts are available at four taxonomic levels and corresponding counts in the data set. (D) Plasmid host prediction performances for 5-mers in AUC, MCC, macro F1, and the confusion matrix. True positive (TP) and true negative (TN) correspond to the correct positive and negative class predictions, respectively. False positive (FP) and false negative (FN) correspond to the false-positive and -negative class predictions, respectively. (E) Plasmid host prediction performances for 8-mers in AUC, MCC, macro F1, and the confusion matrix. True positive (TP) and true negative (TN) correspond to the correct positive and negative class predictions, respectively. False positive (FP) and false negative (FN) correspond to the false-positive and -negative class predictions, respectively. (F) Plasmid host prediction performances for 10-mers in AUC, MCC, macro F1, and the confusion matrix. True positive (TP) and true negative (TN) correspond to the correct positive and negative class predictions, respectively. False positive (FP) and false negative (FN) correspond to the false-positive and -negative class predictions, respectively. (G) Plasmid host prediction performances for 8-mers combined with the plasmid size, GC, and codon usage features in AUC, MCC, macro F1, and the confusion matrix. True positive (TP) and true negative (TN) correspond to the correct positive and negative class predictions, respectively. False positive (FP) and false negative (FN) correspond to the false-positive and -negative class predictions, respectively. (H) Average number of species per cluster. Each cluster was generated based on the *k*-mer query similarity using the given threshold. (I) Plasmid host prediction performances with similarity-clustered plasmids for a given *k*-mer similarity threshold. The plasmid hosts were predicted for 8-mers, and the performances were reported in AUC, MCC, macro F1, and the confusion matrix. True positive (TP) and true negative (TN) correspond to the correct positive and negative class predictions, respectively. False positive (FP) and false negative (FN) correspond to the false-positive and -negative class predictions, respectively. (J) Prediction of plasmid host range(s) with 500-, 1,000-, or 1,500-nucleotide (nt) fragments that were subsequenced into 5-mers. True positive (TP) and true negative (TN) correspond to the correct positive and negative class predictions, respectively. False positive (FP) and false negative (FN) correspond to the false-positive and -negative class predictions, respectively. (K) Prediction of plasmid host range(s) with 500-, 1,000-, or 1,500-nucleotide (nt) fragments that were subsequenced into 8-mers. True positive (TP) and true negative (TN) correspond to the correct positive and negative class predictions, respectively. False positive (FP) and false negative (FN) correspond to the false-positive and -negative class predictions, respectively. (L) The NCBI plasmid IDs and corresponding host names are shown at the order level where the number of plasmids is highest. (M) The NCBI plasmid hosts are available at four taxonomic levels and corresponding counts in the data set. (N) NCBI plasmid host prediction performances for 8-mers in AUC, MCC, macro F1, and the confusion matrix. True positive (TP) and true negative (TN) correspond to the correct positive and negative class predictions, respectively. False positive (FP) and false negative (FN) correspond to the false-positive and -negative class predictions, respectively. (O) Prediction of plasmid host range(s) for the NCBI plasmids with 500-, 1,000-, or 1,500-nucleotide (nt) fragments that were subsequenced into 5-mers. True positive (TP) and true negative (TN) correspond to the correct positive and negative class predictions, respectively. False positive (FP) and false negative (FN) correspond to the false-positive and -negative class predictions, respectively. (P) Prediction of plasmid host range(s) for the NCBI plasmids with 500-, 1,000-, or 1,500-nucleotide (nt) fragments that were subsequenced into 8-mers. True positive (TP) and true negative (TN) correspond to the correct positive and negative class predictions, respectively. False positive (FP) and false negative (FN) correspond to the false-positive and -negative class predictions, respectively. Download Table S1, XLSX file, 0.3 MB. Copyright © 2022 Aytan-Aktug et al. 2022 Aytan-Aktug et al. https://creativecommons.org/licenses/by/4.0/ This content is distributed under the terms of the Creative Commons Attribution 4.0 International license.

To predict the taxonomic label of the host organism, machine learning models were trained using nucleotide *k*-mer counts from the plasmids. The predictions were carried out using 5-, 8-, and 10-mer oligonucleotides, since the short and long *k*-mers might provide different types of information to the models. For example, 5-mers do not usually appear in the plasmid genome uniquely and instead provide the models with information regarding the profile of oligonucleotide frequencies for each plasmid. On the other hand, the longer *k*-mers, such as 8- and 10-mers, are likely to occur uniquely in a given plasmid and offer counts of unique subsequences. Moreover, *k*-mer distributions are subject to changes based on the sequence size.

**FIG 2:**
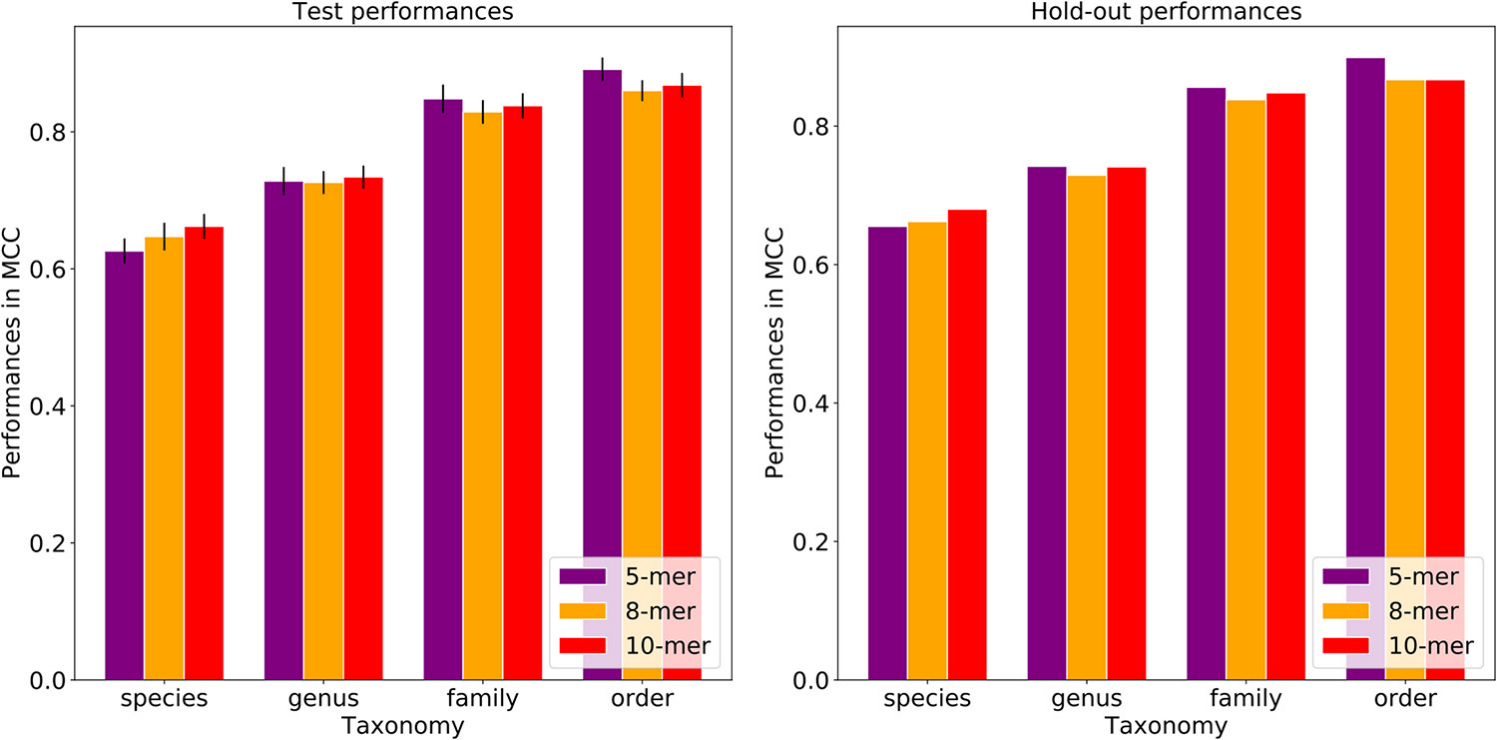
Host prediction performances by *k*-mer size for the test and hold-out data sets. Each bar represents the model performance per taxonomic level. While the test performances were reported with standard deviations, the hold-out performances do not have standard deviations as the five models were combined and a single performance was calculated. The plots show that the prediction performances vary when using different *k*-mer sizes. 5-mers yield the highest MCC at higher taxonomies, while 10-mers yield the highest MCC at lower taxonomies. The model performances generally increased from the species to order level for all the *k*-mer sizes.

Using each *k*-mer size, random forest-based classifiers were built to predict host taxonomy from order to species levels. The model based on the 5-mer counts has a Matthews correlation coefficient (MCC) of 0.655 for predicting the plasmid host species, and this was moderately lower than the 8-mer and 10-mer models, which had MCCs of 0.662 and 0.680, respectively (Fig. 2; Table S1D to F). At the order level, the model performances achieved an MCC of 0.899 for 5-mers and an MCC of 0.867 for 8-mers and 10-mers (Fig. 2; Table S1D to F).

By increasing the *k*-mer size from 5 to 10, the prediction performances increased 3.8% in MCC at the species level but decreased 3.6% at the order level, although the fluctuations in the performances of both sets are not statistically significant based on the paired *t* test (*P* values of 0.404 and 0.883, which are greater than the significance threshold of 0.05). To limit computational needs, we used the 8-mers to build input matrices for all subsequent analyses. Overall, the plasmid host prediction models have low sensitivity (true-positive rate) and high specificity (true negative rate). The lowest sensitivity was detected at the species level compared to other taxonomy levels, where sensitivity falls into the range between 0.493 and 0.761.

**FIG 3:**
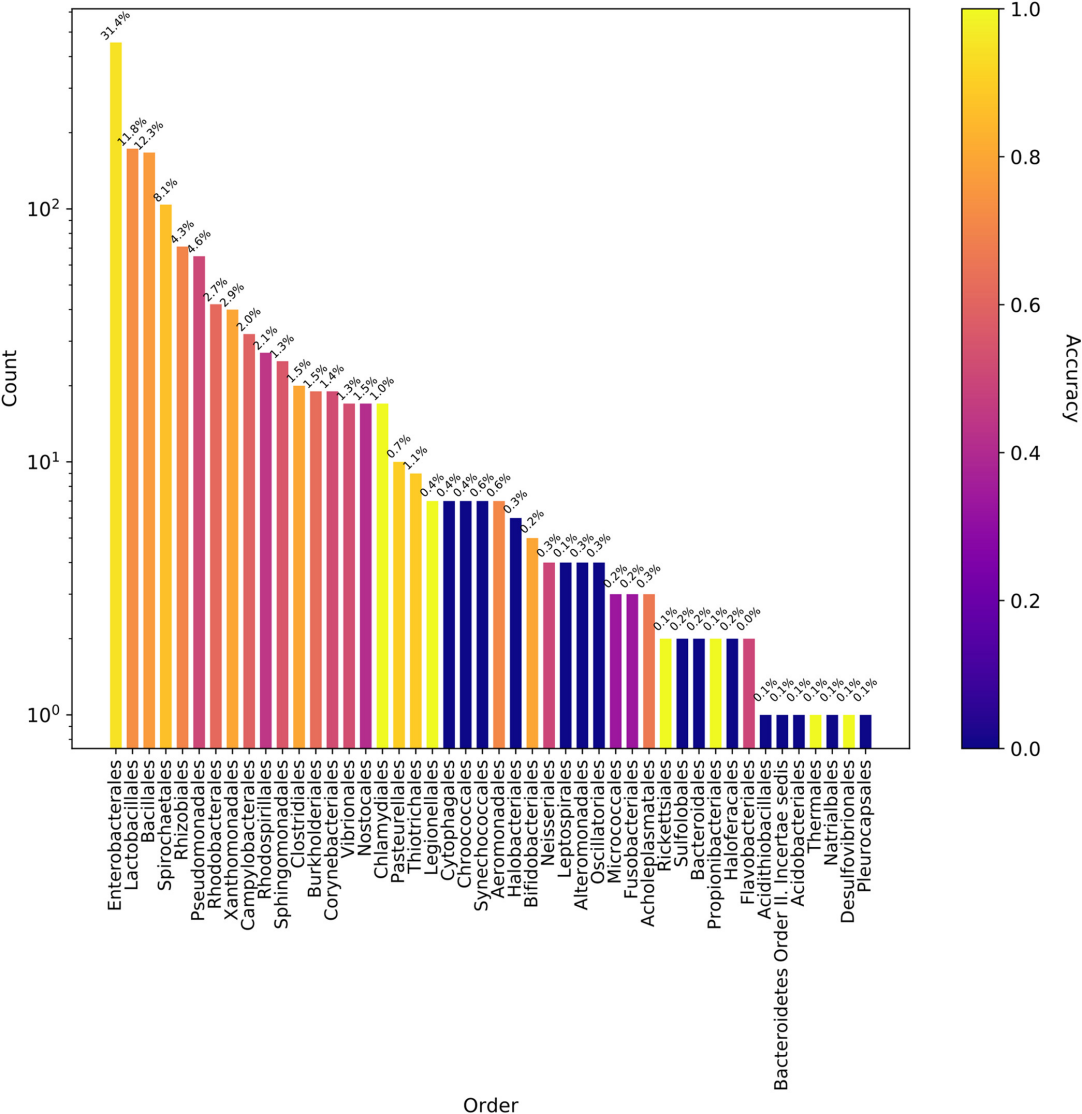
Model accuracy for the PATRIC plasmids tested with the whole model. Each bar shows the number of bacterial orders in the hold-out data, and the corresponding model accuracy was color coded. The percentage on the top of each bars shows the percentage of bacterial orders in the training data.

The ratio of the false-negative predictions was increased inversely by the presence of the hosts in the input data (Fig. 3), moreover, this correlation was significant at the species level (Spearman’s correlation coefficient of 0.545 and *P* value of 0.00, which is less than the significance threshold of 0.05). These findings suggest that the host classification becomes more challenging at the species level, and the model performances improve proportionally to the host representations in the training data. In addition to the false negatives, false-positive predictions made by the model (see Fig. S3 and S4 in the supplemental material) were frequently phylogenetically close to the actual hosts. For instance, the model frequently predicted Escherichia coli hosts instead of Salmonella enterica and Klebsiella pneumoniae, where all of them belong to the *Enterobacteriaceae.*

10.1128/mSystems.01180-21.3 FIG S3 False-positive predictions in the PATRIC hold-out data set. The heat map demonstrates the false predictions by the plasmid host prediction model in the *x* axis and the actual hosts in the *y* axis. The coloring scheme represents the number of times an actual host was predicted as another host. Download FIG S3, PDF file, 1.0 MB. Copyright © 2022 Aytan-Aktug et al. 2022 Aytan-Aktug et al. https://creativecommons.org/licenses/by/4.0/ This content is distributed under the terms of the Creative Commons Attribution 4.0 International license.

10.1128/mSystems.01180-21.4 FIG S4 False positives predicted by the model in addition to the actual hosts. The heat map demonstrates the false predictions in the *x* axis and the actual hosts in the *y* axis. The coloring scheme represents the number of times an actual host was predicted as another host. Download FIG S4, PDF file, 0.1 MB. Copyright © 2022 Aytan-Aktug et al. 2022 Aytan-Aktug et al. https://creativecommons.org/licenses/by/4.0/ This content is distributed under the terms of the Creative Commons Attribution 4.0 International license.

In an attempt to improve the 8-mer model performances, we combined the *k*-mer frequencies with the information in nucleotide compositions of plasmid sequences, including plasmid size, GC content, and codon usage. These additional features yielded an approximately 0.6% to 1.6% increase in the MCCs of the models (see Fig. S2 and Table S1G in the supplemental material); however, this improvement was not statistically significant based on the paired *t* test (*P* value of 0.892, which is greater than the significance threshold of 0.05). Therefore, the following analyses were carried out without these additional features.

10.1128/mSystems.01180-21.2 FIG S2 The contribution of the additional features to the plasmid host range model trained with the 8-mers. The 8-mer counts were combined with additional features, such as plasmid length (size), GC content (gc), and codon usage (aa). The contribution of the additional features (colored purple) was demonstrated by comparing the baseline model (colored orange) in MCC. The prediction performances slightly increased by combining the 8-mer counts with the additional features. Download FIG S2, PDF file, 0.09 MB. Copyright © 2022 Aytan-Aktug et al. 2022 Aytan-Aktug et al. https://creativecommons.org/licenses/by/4.0/ This content is distributed under the terms of the Creative Commons Attribution 4.0 International license.

**FIG 4:**
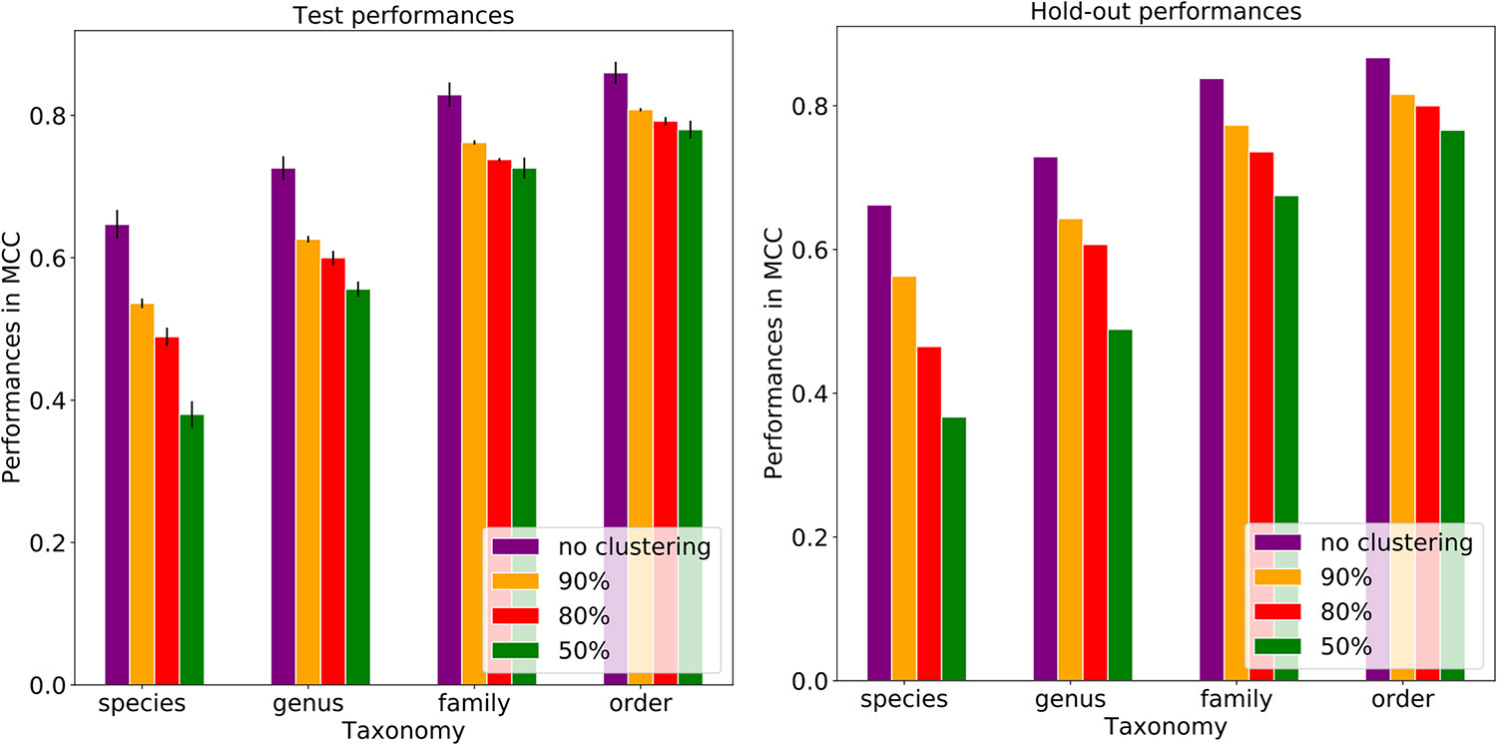
Effects of clustering plasmids at different *k-*mer similarity thresholds on the plasmid host predictions using 8-mers and different taxonomic levels. Each bar represents the model performance per taxonomic level, and each error bar represents the standard deviation across folds. The plot shows the influence of plasmid sequence similarity on prediction performances in MCCs from the species to order level. The plots suggested that the prediction models pick up sequence similarity mostly at lower taxonomic levels. When the dissimilarity was increased between the training, test, and hold-out data sets by applying the 80% *k*-mer similarity threshold, 7.7% to 29.8% losses in MCC performance were observed for the hold-out data.

To understand the impact of plasmid sequence similarity on the model performances, the plasmid genomes were clustered based on the *k*-mer similarity using KMA. The plasmids belonging to the same cluster at a given *k-*mer similarity threshold were kept in the same training, testing, or hold-out data set. When the *k-*mer similarity decreased to 80%, thus making the clusters more inclusive, the model performances decreased in MCC between 7.7% and 29.8%, depending on the taxonomic level (Fig. 4; Table S1H and I). The performance decrease shows that the similarity has an effect on the host predictions, especially at the lower taxonomic levels, although the model can still be generalized to distant sequences.

### Plasmid host predictions with random fragments.

**FIG 5:**
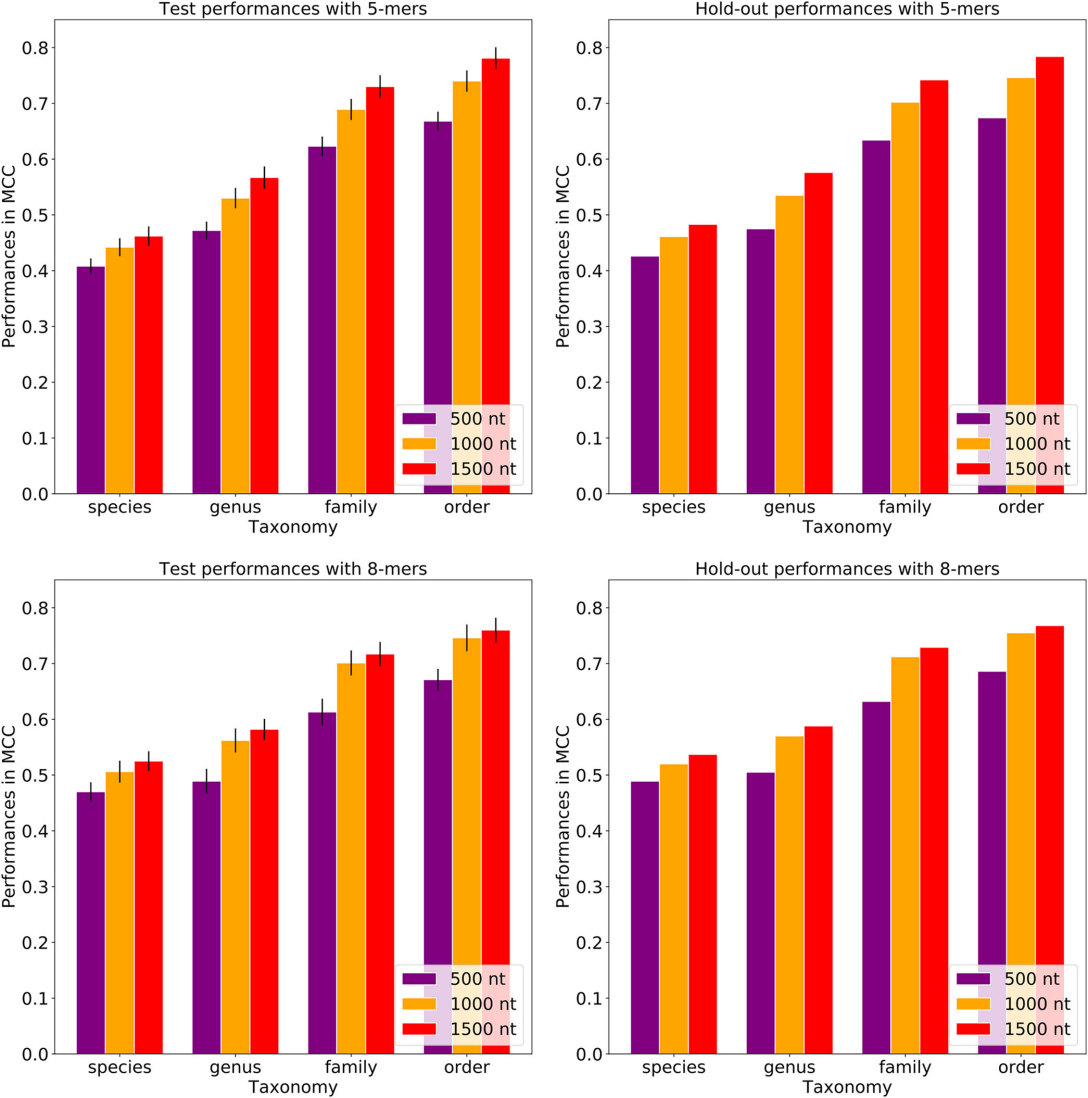
Fragment model performances for the 5-mer and 8-mer models. The fragment models were trained with either 500-, 1,000-, or 1,500-nucleotide (nt) fragments that were subsampled from the PATRIC plasmids. The bar plots show the test and hold-out performances for the 5-mers and 8-mers in MCC. The error bars represent standard deviations. The best-performing model was trained with the 1,500-nucleotide fragments using 8-mers.

Due to the fragmented nature of plasmid assemblies, which results from the difficulty in assembling plasmids from the short reads, we wanted to develop random forest models that can make predictions from incomplete sequences. To do that, we trained and tested our plasmid host prediction models with random fragments of plasmid sequences. Fragments of 500, 1,000, or 1,500 nucleotides were randomly sampled from each assembled plasmid sequence over 10 rounds. By sampling multiple times, we attempted to introduce various regions of the plasmid sequences to the models. The fragment model that was trained with the 500-nucleotide fragments using 5-mers reached MCCs of 0.426 for the species model and 0.674 for the order model (Fig. 5; Table S1J). When the same fragments were subsampled into 8-mers, the species-level model had MCCs of 0.489 and 0.686 MCC for the species and order levels, respectively (Fig. 5; Table S1K). By increasing the fragment size from 500 to 1,000 nucleotides, the model performances increased 8.2% to 10.7% in MCCs with the 5-mers and 6.3% to 10.1% in MCCs with the 8-mers (Fig. 4 and 5; Table S1J to K). When the fragment size increased from 1,000 nucleotides to 1,500 nucleotides, the model performances increased 4.8% to 5.1% in MCCs with the 5-mers and 3.3% to 1.7% in MCCs with the 8-mers (Fig. 4 and 5; Table S1J to K). The fragment models reached their highest performances using 1,500-nucleotide fragments and 8-mers as the features, where the MCCs were 0.537 and 0.768 for the order and species models, respectively (Fig. 5; Table S1K).

### Validation of the plasmid host prediction model with the NCBI validation data set.

**FIG 6:**
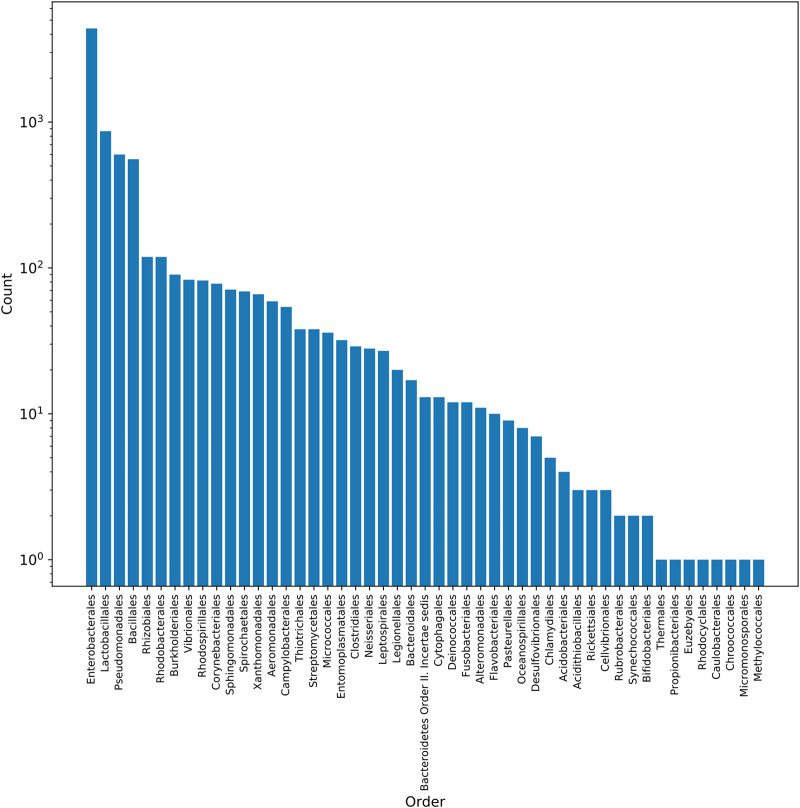
Plasmid host distribution in the NCBI validation data set. The validation data set was dominated by the *Enterobacterales*, *Lactobacillales*, and *Pseudomonadales*, which make up 76% of the NCBI plasmid hosts.

To validate the plasmid host prediction models, we used plasmids in the NCBI RefSeq collection that are not present in our training, test, or hold-out data sets. Overall, 7,670 bacterial plasmid sequences with taxonomic metadata were included in the analysis (Table S1L and M). As in the PATRIC database, the NCBI validation data are also dominated by the few major orders, such as *Enterobacterales*, *Lactobacillales*, and *Pseudomonadales*, which make up approximately 76% of the data set (Fig. 6).

**FIG 7:**
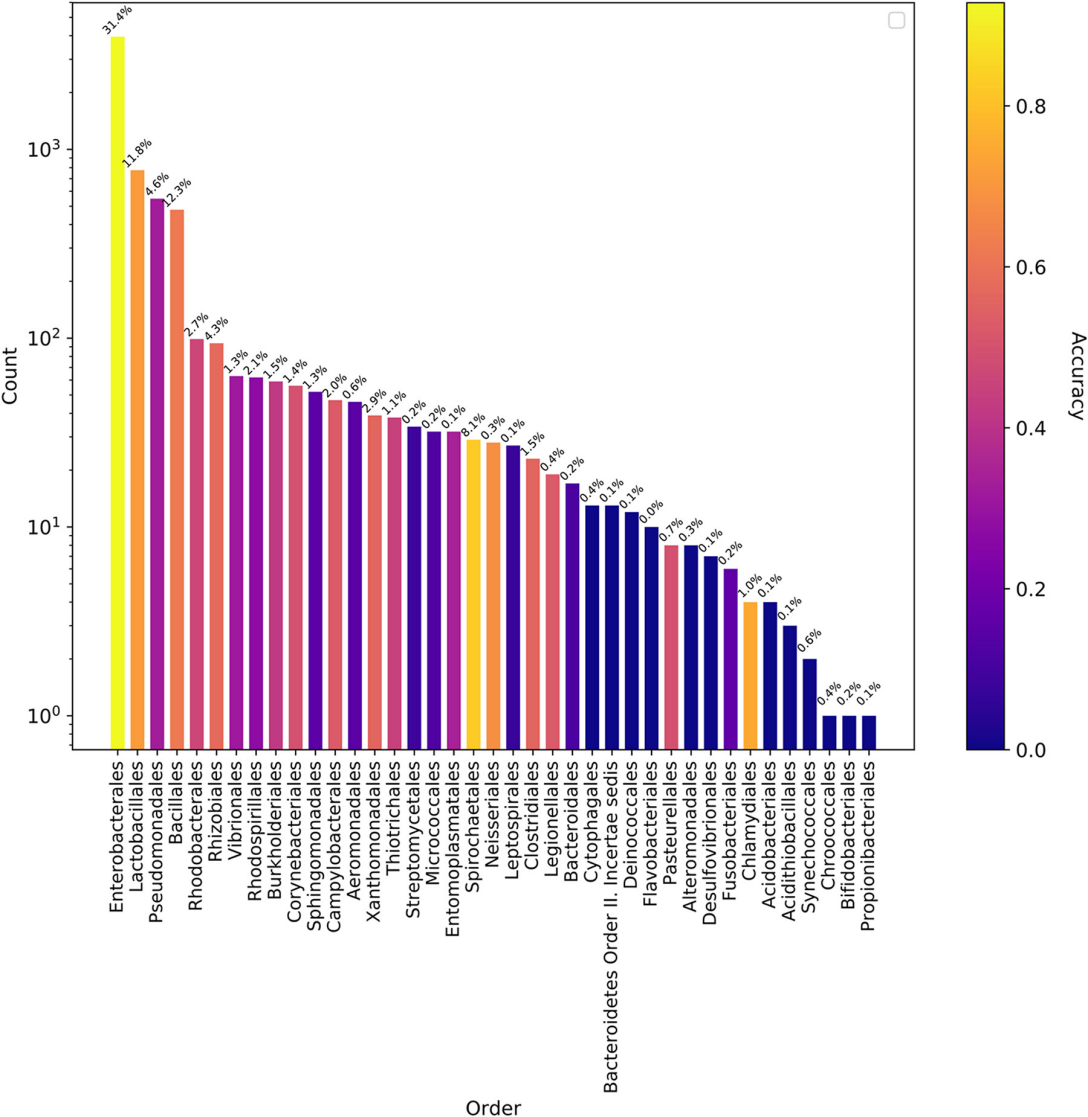
Model accuracy for the NCBI plasmids tested with the whole model that was trained with the PATRIC data set. Each bar shows the number of bacterial orders in the validation data, and the corresponding model accuracy was color coded. The plot showed that the accuracy of the models changed roughly according to the availability of the host organisms in the training data, which is indicated on top of the bars.

When the whole model (trained with 8-mers of the PATRIC training set) was tested with the NCBI validation data, the ratio of the correct and wrong predictions was determined as shown in Fig. 7. Our plasmid host prediction model has relatively low sensitivity (0.483) and a high specificity (1.0) at the species level (Table S1N), similar to the results shown above. Moreover, when the NCBI validation data were tested with the random model generated by shuffled labels, the model performance dropped to an MCC of 0.028 at the species level. This suggests that even though the sensitivity is low, the model has adequate generalizability, which is far from being random.

Because the NCBI collection contained many short plasmid sequences, we filtered it based on the sequence size. Overall, plasmid sequences of ≥5,000 bp performed 43% better than plasmid sequences of <5,000 bp in terms of MCC at the species level. However, this performance gap was reduced to 1% at the order level. This means that the plasmid host range model accuracy improves with longer plasmid sequences at lower taxonomic levels.

As shown in the predictions for the hold-out data set, the plasmid host prediction model predicted additional hosts for 499 plasmids in the NCBI validation data set (see Fig. S5 in the supplemental material). In the validation data set, the model frequently predicted Escherichia coli as being the host instead of the close relatives Salmonella enterica and Klebsiella pneumoniae: this pattern repeats in the hold-out results.

10.1128/mSystems.01180-21.5 FIG S5 False-positive predictions in the NCBI validation data set. The heat map demonstrates the false predictions in the *x* axis and the actual hosts in the *y* axis. The coloring scheme represents the number of times an actual host was predicted as another host. Download FIG S5, PDF file, 0.8 MB. Copyright © 2022 Aytan-Aktug et al. 2022 Aytan-Aktug et al. https://creativecommons.org/licenses/by/4.0/ This content is distributed under the terms of the Creative Commons Attribution 4.0 International license.

**FIG 8:**
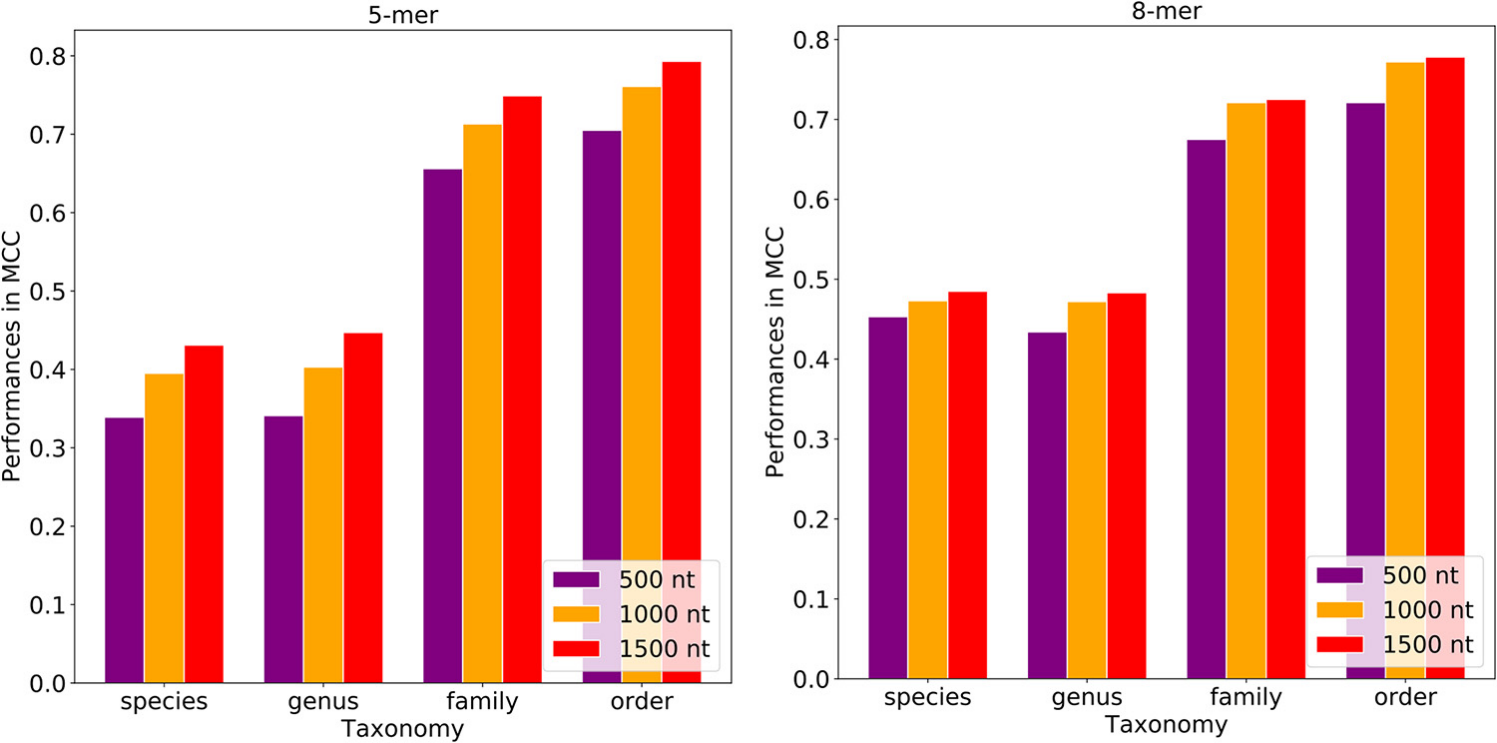
The fragment models were validated with the NCBI plasmids. The fragment models that trained with the 500-, 1,000-, and 1,500-nucleotide (nt) fragments from the PATRIC plasmids were validated with the fragments that were subsampled from the NCBI plasmids. Similar to the hold-out results, the best performance was obtained with the 1,500-nucleotide fragments and 8-mers.

The fragment-based models were also validated using the NCBI data set. The 7,670 NCBI plasmids were randomly subsampled into 500-, 1,000-, and 1,500-nucleotide fragments, and each plasmid was randomly sampled 10 times per fragment size. Similar to the PATRIC results, the fragment models reached the best performances (MCCs of 0.485 at the species level and 0.778 at the order level) for the NCBI validation data with the 1,500-nucleotide fragment size and 8-mers (Fig. 8; Table S1O and P).

### Model interpretation.

To increase our understanding of the host prediction models, we measured the impact of the features on the final decisions. We first found the most important 10 features per model and host and investigated only the features that occurred at least in three out of five models. Second, we detected the origin of these informative 8-mers by mapping them against the plasmid sequences. Finally, we annotated these hits to the gene products. As the 8-mers could appear in the plasmid sequences more than once, the random forest trees could make the decision based on the 8-mer distributions in addition to the absence or presence of the 8-mers. Therefore, the mapping could lead to detection of many irrelevant genes containing the selected 8-mers. In order to reduce the bias in the annotations, we repeated the same analysis with 10-mers. In spite of the multiple occurrences of 8-mers, we detected approximately 80% overlap in the annotated features between 8-mers and 10-mers (data not shown).

The top features that were found important by the majority of the cross-validation models were mostly poorly characterized features, such as polyketide synthase modules and related proteins, hypothetical proteins, mobile element proteins and repeat regions (see Table S2 and Table S3 in the supplemental material). Also, these poor annotations do not provide distinctive information as they were found to be important for each classifier. Therefore, we focused on the well-annotated genes that were only present in a unique host. It was observed that *k*-mers in signal peptides, clustered regulatory interspaced short palindromic repeats (CRISPR) repeats, insertion sequences, transmembrane helices, transcriptional regulators, ABC transporters, and replication origins and replication-related proteins were frequently used in distinguishing the plasmid hosts at the genus and order levels (Table S2 and Table S3).

10.1128/mSystems.01180-21.7 TABLE S2 The feature importance analysis was performed for 10-mers at the genus level. The “Annotation” column shows the gene products that the important 10-mers hit. The “Number of genera” column shows the number of genera in which the corresponding annotation was found important. The names of these genera are shown in the “Genus” column. Download Table S2, XLSX file, 1.1 MB. Copyright © 2022 Aytan-Aktug et al. 2022 Aytan-Aktug et al. https://creativecommons.org/licenses/by/4.0/ This content is distributed under the terms of the Creative Commons Attribution 4.0 International license.

10.1128/mSystems.01180-21.8 TABLE S3 The feature importance analysis was performed for 10-mers at the species level. The “Annotation” column shows the gene products that the important 10-mers hit. The “Number of species” column shows the number of species in which the corresponding annotation was found important. The names of these species are shown in the “Species” column. Download Table S3, XLSX file, 2.4 MB. Copyright © 2022 Aytan-Aktug et al. 2022 Aytan-Aktug et al. https://creativecommons.org/licenses/by/4.0/ This content is distributed under the terms of the Creative Commons Attribution 4.0 International license.

### Comparison to PlasmidFinder and COPLA.

The PlasmidFinder tool uses an alignment-based strategy to identify plasmid sequences and can often provide host information when it is available (3). We used 391 *Enterobacteriaceae* plasmids in the PATRIC validation data set that were not already part of the PlasmidFinder database to compare the accuracy of PlasmidHostFinder to those of PlasmidFinder and COPLA for plasmid host identification. Overall, PlasmidFinder correctly identified the hosts of 336 plasmids, incorrectly identified the hosts of 16 plasmids, and identified no host for 39 plasmids at the species level. The COPLA tool classifies plasmids by assigning them to PTUs, where each PTU establishes a characteristic host range (16, 21). COPLA correctly identified the hosts of 292 plasmids, incorrectly identified the hosts of 12 plasmids and identified no host for 87 plasmids at the species level. It should be noted that COPLA made additional false predictions for 245 out of 292 correctly predicted plasmids. PlasmidHostFinder—for complete sequences—correctly identified the hosts of 229 plasmids, incorrectly identified the hosts of 46 plasmids and identified no host for 116 plasmids at the species level.

In order to compare the accuracies of PlasmidFinder and PlasmidHostFinder for the partial plasmid sequences, we randomly sampled each of the 391 plasmids into 1,500-nucleotide fragments 10 times. Overall, PlasmidFinder was able to identify 347 out of 3,910 fragmented sequences as plasmid derived. However, none of the returned matches contained plasmid host information. On the same set of sequence fragments, PlasmidHostFinder correctly identified the hosts of 1,927 fragmented plasmids, incorrectly identified the hosts of 742 fragmented plasmids, and identified no host for 1,241 fragmented plasmids at the species level. COPLA was not included in this part of the analysis due to the high computational overhead of using the tool.

For the *Enterobacteriaceae* species, the alignment-based PlasmidFinder tool provides more accurate predictions than PlasmidHostFinder when plasmids are near complete. However, when the plasmids are incomplete, the machine learning-based PlasmidHostFinder becomes more advantageous because the presence of specific sequences is required for the correct PlasmidFinder identification.

### The web server.

**FIG 9:**
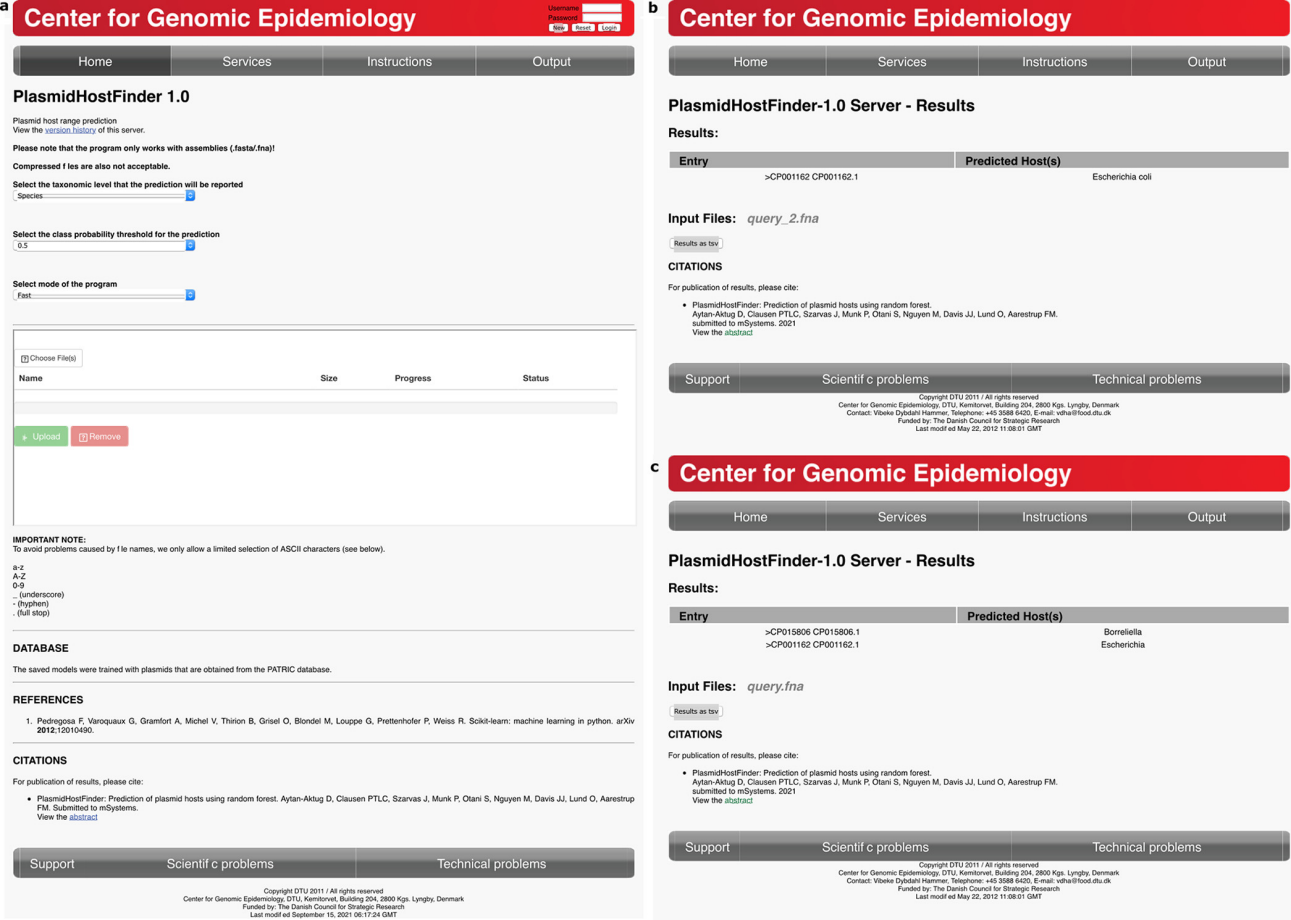
Web server for PlasmidHostFinder. (a) The interface of the PlamidHostFinder web server is shown. The web server runs online after uploading a query file in FASTA format and selecting appropriate parameters for the taxonomic level (species to order), class probability threshold (0.1 to 0.9), and mode of the program (fast or slow). (b) An example of output of the PlasmidHostFinder web server is shown. In this example, the *query_2.fna* file was run using the following parameters: species level, 0.5 class probability threshold, and fast mode. The tool has two kinds of output files: HTML table and downloadable TSV file. While the HTML table includes only the detected plasmid host with at least 0.5 host probability, the TSV file includes the probabilities of all possible hosts at the species level. (c) Another example of the PlasmidHostFinder output. In this example, the *query.fna* file was run using the following parameters: genus level, 0.5 class probability threshold, and fast mode. Differently, this query file includes two contigs in the same file. Thus, the predicted hosts and all of the host probabilities were reported per contig in the HTML table and the TSV file, respectively.

The plasmid host prediction models that were trained using 8-mers from whole plasmid sequences can be used online at the Center for Genomic Epidemiology (https://cge.cbs.dtu.dk/services/PlasmidHostFinder/). This web server tool accepts one FASTA file at a time and provides an output file containing the predicted plasmid host range at the selected taxonomic level from species to order (Fig. 9a). It has two options for running the tool—fast and slow—with various class thresholds (Fig. 9a). The slow mode uses all five cross-validation models to make a final decision on the plasmid host range. The fast mode uses only the first cross-validation model out of five to predict the plasmid host range. Therefore, one can expect to obtain more confident predictions with the slow mode. Once the input file is uploaded and appropriate options are selected, PlasmidHostFinder runs on the CGE server, which is located physically at Technical University of Denmark, Denmark. The web server reports the plasmid host predictions in two formats: an HTML table and a downloadable tab-separated text (TSV) file. In the HTML table, PlasmidHostFinder reports the predicted hosts, defined as having class probabilities that are equal to or greater than the selected class probability threshold, per entry of the given input FASTA file (Fig. 9b and c). Meanwhile, in the TSV file, all possible plasmid hosts and corresponding probabilities are reported, even if the assigned probabilities are below the class probability threshold; this is practical for exploring other possible hosts with different plasmid class thresholds.

## DISCUSSION

In this study, we built random forest models that can predict plasmid hosts and host ranges at taxonomic levels between species and order; these models achieved accuracies from MCCs of 0.662 to 0.867. The model prediction performs better at higher taxonomic levels, with the “order” level being the best. We observed that the *k*-mer size does not have a significant influence on the prediction performances. Among the three *k*-mer sizes, we chose to build our prediction models with 8-mers since the 8-mer provides robust predictions at all taxonomic levels with less computational effort than 10-mers. Moreover, we tried to improve the host range predictions with additional genome features such as plasmid size, GC content, and codon usage, but the increase in the prediction performances was negligible. We validated our models using an independent data set from the NCBI RefSeq. These performances were comparable with our previous test and validation results. In addition, to assess the utility of this approach with partially assembled plasmid sequences, we generated models for 500-, 1,000-, and 1,500-nucleotide fragments, and even the smallest fragments of 500 nucleotides have sufficient information for the identification of plasmid hosts. Overall, the PlasmidHostFinder models were trained using assemblies from short or long reads. Although the online tool supports predictions based on the assumption that the sequence file is based on complete plasmid sequences, in theory, the fragment-based models could be extended to putative plasmid sequence from assembled metagenomic samples using the command line-based tool.

### Machine learning.

We observe that the robustness of the models is dependent on the quantity, quality, and accuracy of the input and output data. In this study, the plasmid host prediction models might suffer from incomplete metadata. The plasmid data and corresponding plasmid hosts were retrieved from the PATRIC database. However, the PATRIC data set is likely to contain some plasmids with incomplete host range information. This issue might have an effect on robustness of the models, but is most likely to have a minor impact due to the relatively large input data set. Nevertheless, some of the false-positive predictions might be the consequence of incomplete metadata. Some of the other false positives might potentially be new discoveries relating to plasmid transmission in diverse hosts, although this should be validated experimentally.

PlasmidHostFinder uses a different set of random forest models per taxonomic level, namely, species, genus, family, and order. The accuracy of models increases going from the species to order levels. These general predictions are a useful approximation when the species-level predictions are not available. This can happen when the host probabilities of a query plasmid remain below the given probability threshold. In addition, predicting the same host at several taxonomy levels provides a way to assess the accuracy of predictions by confirming the predictions at different levels.

Plasmid genomes are extremely plastic (22). Accessory genes vary in their presence or absence from the plasmids, which makes plasmid host prediction a complicated task. In order to understand the impact of the genome similarity on the plasmid host model learning, we clustered the plasmids for a given similarity threshold. By keeping the similar plasmids in the same training, test, or hold-out data sets, the learning from the sequence similarity was minimized since the similar plasmids tend to have the same hosts. This clustering approach caused less accurate results than the baseline model. These results suggest that sequence similarity has an impact on the model learning. Therefore, to boost the model performances, the training data should be updated regularly to increase the input diversity when more plasmid data are available. In addition to the sequence similarity, host-related signals from the relatively conserved regions of the plasmid sequences, such as *rep* or *mob* genes, are learned from the model (Table S2 and Table S3).

The model performances were evaluated using several performance measurements, including area under the curve (AUC), MCC, and macro F1. The AUC and MCC performances were not always correlated and caused different conclusions in some cases, such as in the random forest model with the clustered plasmids. The reason for this discrepancy may be the applied class thresholds. AUC uses a range of thresholds to measure the model performances and does not require a defined class threshold. In contrast to AUC, the MCC and macro F1 calculations require predictions instead of probabilities. Therefore, a defined class probability threshold is needed for conversion of probabilities to predictions. This threshold was set to 0.5 for all the models. However, this threshold might not be the ideal threshold for some of the models, particularly for the imbalanced classes (23). For instance, the species-level prediction model has a lower sensitivity (0.493) than its specificity (1.0). In other words, the model failed to predict some of the hosts (Fig. 3 and 7), and the majority of the failed predictions were the result of having no positive class predicted for the tested plasmid due to no predictions being above or equal to the class probability threshold of 0.5. Therefore, adjusting the class probability threshold could be a way to improve the model.

### False positives or unknown hosts.

The machine learning models have the potential for discovery of unknown correlations between the input features and predicted phenotypes. For example, in previous studies, novel AMR genes were reported using the machine learning models (18, 19). In our case, machine learning might be useful for discovering unknown plasmid hosts by learning the similar genetic patterns assigned to different phenotypes, such as in 99% *k*-mer similar plasmids with different hosts (Table S1H). We explored these potential discoveries (in other words false positives), as in cases where (i) the model was not able to predict the actual hosts, but predicted false positives (Fig. S3), and (ii) the model predicted multiple hosts, including the actual hosts and false positives (Fig. S4). These cases should be investigated further as these could happen due to two reasons: the model might pick up noise, or the falsely predicted host might actually be a host in nature. Thus, a portion of the false positives might be the actual hosts, which were not discovered before, but machine learning gives the opportunity for discovery *in silico*. To prove that they are potential hosts would require *in vitro* experiments to test the stability of the plasmid in these bacteria.

### Fragments.

The fragment-based model performances vary based on the fragment and *k*-mer sizes. We obtained the best performances for the hold-out data set with the 1,500-nucleotide fragments using 8-mers. The fragment size and model performances changed proportionally because the longer fragments provide more information to the models. This might be the consequence of the mosaic nature of plasmids. Genes located on plasmids could originate from different organisms, and random sampling of these acquired genes might cause false predictions. Moreover, as the plasmids were not aligned prior to the fragmentation, the genetic content of fragments subsampled from different plasmids did not match. Therefore, the model might be learning the fragment structures in most of the cases.

### Conclusion.

We built random forest models and incorporated them in the PlasmidHostFinder tool to detect plasmid hosts and host ranges at various taxonomic levels, from species to order, with MCC performance of 0.662 to 0.867. PlasmidHostFinder can detect a diverse range of hosts for 359 species, 174 genera, 93 families, and 50 orders with high accuracy in spite of the mosaic and diverse nature and genetic plasticity of plasmids. The approach described in this study helps to fill a gap in our ability to predict plasmid hosts, particularly in understudied taxa or when plasmid sequences are fragmented.

## MATERIALS AND METHODS

### Data set.

We downloaded all of the available (10,863) plasmids and corresponding metadata from the Pathosystems Resource Integration Center (PATRIC) (24) in September of 2020. Metadata for each plasmid included the origin of the plasmid and other relevant information, such as different database accession numbers, collection dates and places, and genomic lengths and features. Four plasmids did not have host information and were removed. The remaining plasmid host information was reported from genus to strain level by the PATRIC database. In total, 1,662 different genus- and species-level hosts were detected in the plasmid metadata. When less than five plasmids had a host at a given taxonomic level, they were removed. In total, 1,296 underrepresented hosts and corresponding plasmids were removed from the data set to improve the robustness of the models. From the remaining 366 hosts, seven were removed for lacking species annotations. Therefore, we ended up with 8,658 plasmids and 359 corresponding hosts with species-level taxonomy information. The species-level plasmid hosts were assigned to the higher taxonomy levels, such as genus, family, and order, using the NCBI Taxonomy information by the Python ete2 package (version 2.3.10) (25, 26).

### Distance tree.

The diversity of the plasmids was measured using an oligonucleotide *k*-mer-based distance tree. The plasmid sequences were indexed using the KMA tool (version 1.3.9) (27) with the following parameters: -NI and -Sparse TG. Next, the 16-mer Hamming distances were calculated. The distance tree was generated using the CCPhylo tool (version 0.2.2) by the neighbor-joining method (28). The distance tree was visualized using iTOL (version 4) (29).

### *k*-mer counts.

The plasmid genomes were subsampled using overlapping *k*-length nucleotides, and the occurrence of every *k*-mer was counted. *k*-mer counting is a well-studied method for analyzing sequence data (30). The subsequence size *k* is a critical parameter as the subsequences yield various pieces of information, depending on the size. While short *k*-mers provide information regarding the sequence content, long *k*-mers are informative in detection of unique sequence patterns. We analyzed plasmid genomes using three different *k*-mer sizes: 5, 8, and 10 nucleotides. Counts were calculated using KMC (version 3.0.0) (31) with the following parameters -fm, -ci1, -cs1677215. These parameters inform the tool regarding the input data format and the minimum and maximum thresholds for the *k*-mer occurrences, respectively.

### Detection of duplicates.

To eliminate possible duplicates from the plasmid collections, we compared the 8-mer counts of the plasmids to each other. Plasmid pairs with identical 8-mer counts were treated as duplicates and merged in the data set. When the pairs had different host information, the additional hosts were incorporated in the metadata. In the 3,658 plasmids, 139 duplicates were detected and excluded from the collection. The remaining 3,519 plasmids were used for generation of machine learning models.

### Sequence length, GC content, and codon usage calculation.

To capture the plasmid genome characteristics, we calculated the total length of the sequence, GC content, and codon usage. Sequence length was calculated by taking all nucleotides into consideration, including ambiguous bases. GC content was calculated by taking the ratio of the total number of cytosine and guanine nucleotides to all nucleotides. Codon usage was determined as the relative frequencies of amino acids in a coding region, which was detected using Prodigal (version 2.6.3) with default parameters (32).

### Model generation and cross-validation.

For each *k*-mer size, a matrix was generated from *k*-mer counts where the rows represent each plasmid, the columns represent each *k*-mer, and the entries represent the *k*-mer count. Additionally, a merged matrix was generated by combining the 8-mer count matrix with the genome length, GC content, and codon usage information.

In this study, we generated multilabel models that are able to predict multiple hosts per plasmid. Each label corresponds to a plasmid host and encodes a binary value, with “1” corresponding to being a host. These plasmid hosts were predicted at different taxonomy levels, such as species, genus, family, and order, where we built separate models per taxonomic level. We used random forest to build the classifiers, which provides robust and interpretable predictions based on decision trees and has been explored in many other classification studies (18, 33–35).

Model parameter tuning and validation were performed using the plasmid data where the data were split into training, testing, and hold-out data sets. The training and testing data sets were used for parameter tuning, and the hold-out data set was used for monitoring possible overfitting. Random forest was implemented using *ensemble.RandomForestClassifier* from the Python Scikit-learn package (version 0.20.4) (36). The model parameters were tuned in a 5-fold cross-validation using the random grid search method from Scikit-learn, which iterated 100 times; n_estimators, max_features, max_depth, min_samples_split, min_samples_leaf, and bootstrap were the parameters tuned (Table S1A). These parameters were responsible for the number of trees in the forest, the number of features used for each split, the maximum depth of the decision tree, the minimum number of samples for splitting a node, the minimum number of samples required for the leaf, and bootstrapping of samples, respectively. Tuning was conducted using an 8-mer matrix at the genus level and then applied to the other taxonomic levels and *k*-mer sizes. The detected optimal parameters were n_estimators = 1,000, max_features = “auto” (which is the square root of the number of features), max_depth = 50, min_samples_split = 2, min_samples_leaf = 1, and bootstrap = False. The class_weight parameter was set to “balanced” to weight the inputs based on the class frequencies to prevent the biased predictions due to the imbalanced classes. The random forest model was utilized with the *multiclass.OneVsRestClassifier* from the Python Scikit-learn (version 0.20.4) package (36), which trains one model per label and improves the interpretability of the models.

Using the tuned parameters, the random forest model was trained and tested five times using the *k*-fold cross-validation method, where different data sets were tested each time. The ensembled cross-validation model was applied on the hold-out data set, which was not part of the training or testing. Model performances were measured with the area under curve (AUC), macro F1 score, Matthews correlation coefficient (MCC), and the confusion matrix by using Scikit-learn (36). Sensitivity and specificity were also calculated from the confusion matrices to measure the ability of model to identify hosts. The class probability threshold of 0.5 was applied to calculate the performances of macro F1, MCC, and the confusion matrix. Since it is a multilabel problem, all of the predictions for all possible labels were collected into one data container, and one prediction performance was calculated per model instead of the number of labels. The test and hold-out data set performances were reported where the test performances were calculated by averaging five model performances and reported with standard deviations, and the hold-out performances were calculated on the average of the five model probabilities.

To test whether the plasmid host model predictions were significantly different, a *t* test was performed using *stats.ttest_*ind from SciPy (version 1.2.2) (37). Moreover, possible correlations were detected with Spearman’s correlation coefficient using *stats.spearmanr* from SciPy (version 1.2.2).

### Clustering plasmids.

Since similar plasmids are likely to be hosted by the same organisms, we clustered the plasmids based on *k*-mer sequence similarity using KMA index (version: 1.3.9) with the following parameters: -k16, -Sparse, and -NI (27). KMA clusters the sequence for a given similarity threshold using 16-mers and the Hobohm-1 algorithm (38). We clustered the plasmids using three different *k*-mer query and template similarity thresholds at 90%, 80%, and 50%. By dividing the clusters into training, testing, and hold-out sets, similar plasmids were kept in the same partitions. This allowed us to see how the models generalized with a set of new, genetically distant plasmids.

### Random fragments.

Partial sequences might be informative for predicting hosts and better reflect actual data in incomplete plasmid assemblies. Therefore, random fragments of 500, 1,000, and 1,500 nucleotides were subsampled from each plasmid sequence to build prediction models from the partial sequences. The subsampling process was randomly replicated 10 times for each plasmid. Plasmids shorter than the given fragment size were excluded from the study, and separate models were built per fragment size. Matrix files and models were constructed as described above, using *k*-mers that were 5 and 8 nucleotides in length. 10-mers were not utilized due to the heavy computational requirements.

### External validation of the plasmid host prediction models.

The plasmid host models that were trained with the PATRIC plasmids at four different taxonomic levels were further validated using plasmids from the NCBI RefSeq database (39). A total set of 30,349 NCBI plasmids were downloaded from the NCBI RefSeq database and filtered. NCBI offers a larger plasmid collection than PATRIC, yet some of the plasmids are identical. Therefore, only the plasmids that had not yet been integrated into PATRIC as of January 2021 (i.e., plasmids that only exist in the NCBI RefSeq database) were included. Further, we eliminated duplicates from the NCBI validation data set by comparing *k*-mer counts and filtered based on the source organism, completeness, and NCBI’s automatic taxonomy check. Moreover, plasmids with labels that are not included in the PATRIC training data were further removed from the NCBI validation data. The remaining plasmids with species-level host information recognized by NCBI Taxonomy were tested against the plasmid host models that were computed from the PATRIC collection. The validation performances were reported in AUC, macro F1, MCC, and the confusion matrix. The class probability threshold of 0.5 was applied to calculate the performances of macro F1, MCC, and the confusion matrix.

### Feature importance.

To explore the feature contributions to the random forest models, the model features (8-mers or 10-mers) were sorted based on their impacts on the final predictions by the *feature_importances_* attribute of Scikit-learn. For each host, the top 10 important features were detected and considered important if they ranked in the top 10 in three out of five cross-validation models. Exact coordinates of these selected *k*-mers were found by mapping the forward and reverse complements of the *k-*mers against the plasmid sequences in the training set, using the custom Python scripts. The corresponding genes to these *k*-mer hits were identified using the PATRIC annotation service (40).

### Comparison to PlasmidFinder and COPLA.

We compared our model performance to PlasmidFinder and COPLA for the *Enterobacteriaceae* species that are present in the PATRIC hold-out data set (3, 16, 21, 41). Moreover, the hold-out plasmids that present in the PlasmidFinder database were excluded from this comparison. The PlasmidFinder (version 2.1.1) and COPLA (version 1.0) tools were run with the default parameters, except the minimum identity threshold parameter of PlasmidFinder, which was set to 0.8. The database for PlasmidFinder was downloaded in July 2021.

### Data availability.

The Python 2.7.15 scripts that used in this study are available on Bitbucket (https://bitbucket.org/deaytan/plasmid-host-prediction/src/master/). The web server is available on Center for Genomic Epidemiology (https://cge.cbs.dtu.dk/services/PlasmidHostFinder-1.0/). All the PATRIC and the NCBI RefSeq sequences and corresponding metadata can be accessed through the PATRIC (https://www.patricbrc.org) and NCBI (ftp://ftp.ncbi.nlm.nih.gov/refseq/release/plasmid/) resources, respectively.

## References

[1] Rodríguez-Beltrán J, DelaFuente J, León-Sampedro R, MacLean RC, San Millán Á. 2021. Beyond horizontal gene transfer: the role of plasmids in bacterial evolution. Nat Rev Microbiol 19:347–359. doi:10.1038/s41579-020-00497-1. 33469168

[2] Orlek A, Phan H, Sheppard AE, Doumith M, Ellington M, Peto T, Crook D, Walker AS, Woodford N, Anjum MF, Stoesser N. 2017. Ordering the mob: insights into replicon and MOB typing schemes from analysis of a curated dataset of publicly available plasmids. Plasmid 91:42–52. doi:10.1016/j.plasmid.2017.03.002. 28286183 PMC5466382

[3] Carattoli A, Zankari E, García-Fernández A, Voldby Larsen M, Lund O, Villa L, Møller Aarestrup F, Hasman H. 2014. *In silico* detection and typing of plasmids using PlasmidFinder and plasmid multilocus sequence typing. Antimicrob Agents Chemother 58:3895–3903. doi:10.1128/AAC.02412-14. 24777092 PMC4068535

[4] Lozano C, García-Migura L, Aspiroz C, Zarazaga M, Torres C, Aarestrup FM. 2012. Expansion of a plasmid classification system for Gram-positive bacteria and determination of the diversity of plasmids in *Staphylococcus aureus* strains of human, animal, and food origins. Appl Environ Microbiol 78:5948–5955. doi:10.1128/AEM.00870-12. 22685157 PMC3406130

[5] Orlek A, Stoesser N, Anjum MF, Doumith M, Ellington MJ, Peto T, Crook D, Woodford N, Walker AS, Phan H, Sheppard AE. 2017. Plasmid classification in an era of whole-genome sequencing: application in studies of antibiotic resistance epidemiology. Front Microbiol 8:182. doi:10.3389/fmicb.2017.00182. 28232822 PMC5299020

[6] San Millan A, MacLean RC. 2017. Fitness costs of plasmids: a limit to plasmid transmission. Microbiol Spectr doi:10.1128/microbiolspec.MTBP-0016-2017. PMC11687550 28944751

[7] Unterholzner SJ, Poppenberger B, Rozhon W. 2013. Toxin-antitoxin systems: biology, identification, and application. Mob Genet Elements 3:e26219. doi:10.4161/mge.26219. 24251069 PMC3827094

[8] Bello-López JM, Cabrero-Martínez OA, Ibáñez-Cervantes G, Hernández-Cortez C, Pelcastre-Rodríguez LI, Gonzalez-Avila LU, Castro-Escarpulli G. 2019. Horizontal gene transfer and its association with antibiotic resistance in the genus *Aeromonas spp*. Microorganisms 7:363. doi:10.3390/microorganisms7090363. PMC6780555 31540466

[9] Jain A, Srivastava P. 2013. Broad host range plasmids. FEMS Microbiol Lett 348:87–96. doi:10.1111/1574-6968.12241. 23980652

[10] Suzuki H, Yano H, Brown CJ, Top EM. 2010. Predicting plasmid promiscuity based on genomic signature. J Bacteriol 192:6045–6055. doi:10.1128/JB.00277-10. 20851899 PMC2976448

[11] Robertson J, Bessonov K, Schonfeld J, Nash JHE. 2020. Universal whole-sequence-based plasmid typing and its utility to prediction of host range and epidemiological surveillance. Microb Genom doi:10.1099/mgen.0.000435. PMC7660255 32969786

[12] Suzuki H, Brown CJ, Top EM. 2018. Genomic signature analysis to predict plasmid host range, p 458–464. *In* Wells RD, Bond JS, Klinman J, Masters BSS (ed), Molecular life sciences: an encyclopedic reference. Springer, New York, NY.

[13] Suzuki H, Sota M, Brown CJ, Top EM. 2008. Using Mahalanobis distance to compare genomic signatures between bacterial plasmids and chromosomes. Nucleic Acids Res 36:e147. doi:10.1093/nar/gkn753. 18953039 PMC2602791

[14] Norberg P, Bergstrom M, Jethava V, Dubhashi D, Hermansson M. 2011. The IncP-1 plasmid backbone adapts to different host bacterial species and evolves through homologous recombination. Nat Commun 2:268. doi:10.1038/ncomms1267. 21468020 PMC3104523

[15] Krawczyk PS, Lipinski L, Dziembowski A. 2018. PlasFlow: predicting plasmid sequences in metagenomic data using genome signatures. Nucleic Acids Res 46:e35. doi:10.1093/nar/gkx1321. 29346586 PMC5887522

[16] Redondo-Salvo S, Bartomeus-Penalver R, Vielva L, Tagg KA, Webb HE, Fernandez-Lopez R, de la Cruz F. 2021. COPLA, a taxonomic classifier of plasmids. BMC Bioinformatics 22:390. doi:10.1186/s12859-021-04299-x. 34332528 PMC8325299

[17] Xu C, Jackson SA. 2019. Machine learning and complex biological data. Genome Biol 20:76. doi:10.1186/s13059-019-1689-0. 30992073 PMC6469083

[18] Aytan-Aktug D, Clausen PTLC, Bortolaia V, Aarestrup FM, Lund O. 2020. Prediction of acquired antimicrobial resistance for multiple bacterial species using neural networks. mSystems 5:e00774-19. doi:10.1128/mSystems.00774-19. PMC6977075 31964771

[19] Kavvas ES, Catoiu E, Mih N, Yurkovich JT, Seif Y, Dillon N, Heckmann D, Anand A, Yang L, Nizet V, Monk JM, Palsson BO. 2018. Machine learning and structural analysis of *Mycobacterium tuberculosis* pan-genome identifies genetic signatures of antibiotic resistance. Nat Commun 9:4306. doi:10.1038/s41467-018-06634-y. 30333483 PMC6193043

[20] Ruppe E, Ghozlane A, Tap J, Pons N, Alvarez AS, Maziers N, Cuesta T, Hernando-Amado S, Clares I, Martinez JL, Coque TM, Baquero F, Lanza VF, Maiz L, Goulenok T, de Lastours V, Amor N, Fantin B, Wieder I, Andremont A, van Schaik W, Rogers M, Zhang X, Willems RJL, de Brevern AG, Batto JM, Blottiere HM, Leonard P, Lejard V, Letur A, Levenez F, Weiszer K, Haimet F, Dore J, Kennedy SP, Ehrlich SD. 2019. Prediction of the intestinal resistome by a three-dimensional structure-based method. Nat Microbiol 4:112–123. doi:10.1038/s41564-018-0292-6. 30478291

[21] Redondo-Salvo S, Fernandez-Lopez R, Ruiz R, Vielva L, de Toro M, Rocha EPC, Garcillan-Barcia MP, de la Cruz F. 2020. Pathways for horizontal gene transfer in bacteria revealed by a global map of their plasmids. Nat Commun 11:3602. doi:10.1038/s41467-020-17278-2. 32681114 PMC7367871

[22] Kostlbacher S, Collingro A, Halter T, Domman D, Horn M. 2021. Coevolving plasmids drive gene flow and genome plasticity in host-associated intracellular bacteria. Curr Biol 31:346–357.e3. doi:10.1016/j.cub.2020.10.030. 33157023 PMC7846284

[23] Saito T, Rehmsmeier M. 2015. The Precision-Recall plot is more informative than the ROC plot when evaluating binary classifiers on imbalanced datasets. PLoS One 10:e0118432. doi:10.1371/journal.pone.0118432. 25738806 PMC4349800

[24] Davis JJ, Wattam AR, Aziz RK, Brettin T, Butler R, Butler RM, Chlenski P, Conrad N, Dickerman A, Dietrich EM, Gabbard JL, Gerdes S, Guard A, Kenyon RW, Machi D, Mao C, Murphy-Olson D, Nguyen M, Nordberg EK, Olsen GJ, Olson RD, Overbeek JC, Overbeek R, Parrello B, Pusch GD, Shukla M, Thomas C, VanOeffelen M, Vonstein V, Warren AS, Xia F, Xie D, Yoo H, Stevens R. 2020. The PATRIC Bioinformatics Resource Center: expanding data and analysis capabilities. Nucleic Acids Res 48:D606–D612. doi:10.1093/nar/gkz943. 31667520 PMC7145515

[25] Huerta-Cepas J, Dopazo J, Gabaldón T. 2010. ETE: a python environment for tree exploration. BMC Bioinformatics 11:24. doi:10.1186/1471-2105-11-24. 20070885 PMC2820433

[26] Schoch CL, Ciufo S, Domrachev M, Hotton CL, Kannan S, Khovanskaya R, Leipe D, McVeigh R, O’Neill K, Robbertse B, Sharma S, Soussov V, Sullivan JP, Sun L, Turner S, Karsch-Mizrachi I. 2020. NCBI Taxonomy: a comprehensive update on curation, resources and tools. Database (Oxford) 2020:baaa062. doi:10.1093/database/baaa062. 32761142 PMC7408187

[27] Clausen PTLC, Aarestrup FM, Lund O. 2018. Rapid and precise alignment of raw reads against redundant databases with KMA. BMC Bioinformatics 19:307. doi:10.1186/s12859-018-2336-6. 30157759 PMC6116485

[28] Hallgren MB, Overballe-Petersen S, Lund O, Hasman H, Clausen P. 2021. MINTyper: an outbreak-detection method for accurate and rapid SNP typing of clonal clusters with noisy long reads. Biol Methods Protoc 6:bpab008. doi:10.1093/biomethods/bpab008. 33981853 PMC8106442

[29] Letunic I, Bork P. 2019. Interactive Tree Of Life (iTOL) v4: recent updates and new developments. Nucleic Acids Res 47:W256–W259. doi:10.1093/nar/gkz239. 30931475 PMC6602468

[30] Sievers A, Bosiek K, Bisch M, Dreessen C, Riedel J, Froß P, Hausmann M, Hildenbrand G. 2017. K-mer content, correlation, and position analysis of genome DNA sequences for the identification of function and evolutionary features. Genes 8:122. doi:10.3390/genes8040122. PMC5406869 28422050

[31] Kokot M, Dlugosz M, Deorowicz S. 2017. KMC 3: counting and manipulating k-mer statistics. Bioinformatics 33:2759–2761. doi:10.1093/bioinformatics/btx304. 28472236

[32] Hyatt D, Chen G-L, LoCascio PF, Land ML, Larimer FW, Hauser LJ. 2010. Prodigal: prokaryotic gene recognition and translation initiation site identification. BMC Bioinformatics 11:119. doi:10.1186/1471-2105-11-119. 20211023 PMC2848648

[33] Pataki BÁ, Matamoros S, van der Putten BCL, Remondini D, Giampieri E, Aytan-Aktug D, Hendriksen RS, Lund O, Csabai I, Schultsz C, Matamoros S, Janes V, Hendriksen RS, Lund O, Clausen P, Aarestrup FM, Koopmans M, Pataki B, Visontai D, Stéger J, Szalai-Gindl JM, Csabai I, Pakseresht N, Rossello M, Silvester N, Amid C, Cochrane G, Schultsz C, Pradel F, Westeel E, Fuchs S, Kumar SM, Xavier BB, Ngoc MN, Remondini D, Giampieri E, Pasquali F, Petrovska L, Ajayi D, Nielsen EM, Trung NV, Hoa NT, Ishii Y, Aoki K, McDermott P, SPS COMPARE ML-AMR Group. 2020. Understanding and predicting ciprofloxacin minimum inhibitory concentration in *Escherichia coli* with machine learning. Sci Rep 10:15026. doi:10.1038/s41598-020-71693-5. 32929164 PMC7490380

[34] Breiman L. 2001. Random forests. Machine Learning 45:5–32. doi:10.1023/A:1010933404324.

[35] Sarica A, Cerasa A, Quattrone A. 2017. Random Forest algorithm for the classification of neuroimaging data in Alzheimer's disease: a systematic review. Front Aging Neurosci 9:329. doi:10.3389/fnagi.2017.00329. 29056906 PMC5635046

[36] Pedregosa F, Varoquaux G, Gramfort A, Michel V, Thirion B, Grisel O, Blondel M, Louppe G, Prettenhofer P, Weiss R. 2012. Scikit-learn: machine learning in python. arXiv 12010490. https://arxiv.org/abs/1201.0490v2.

[37] Virtanen P, Gommers R, Oliphant TE, Haberland M, Reddy T, Cournapeau D, Burovski E, Peterson P, Weckesser W, Bright J, van der Walt SJ, Brett M, Wilson J, Millman KJ, Mayorov N, Nelson ARJ, Jones E, Kern R, Larson E, Carey CJ, Polat İ, Feng Y, Moore EW, VanderPlas J, Laxalde D, Perktold J, Cimrman R, Henriksen I, Quintero EA, Harris CR, Archibald AM, Ribeiro AH, Pedregosa F, van Mulbregt P, Vijaykumar A, Bardelli AP, Rothberg A, Hilboll A, Kloeckner A, Scopatz A, Lee A, Rokem A, Woods CN, Fulton C, Masson C, Häggström C, Fitzgerald C, Nicholson DA, Hagen DR, Pasechnik DV, SciPy 1.0 Contributors. 2020. SciPy 1.0: fundamental algorithms for scientific computing in Python. Nat Methods 17:261–272. doi:10.1038/s41592-019-0686-2. 32015543 PMC7056644

[38] Hobohm U, Scharf M, Schneider R, Sander C. 1992. Selection of representative protein data sets. Protein Sci 1:409–417. doi:10.1002/pro.5560010313. 1304348 PMC2142204

[39] O'Leary NA, Wright MW, Brister JR, Ciufo S, Haddad D, McVeigh R, Rajput B, Robbertse B, Smith-White B, Ako-Adjei D, Astashyn A, Badretdin A, Bao Y, Blinkova O, Brover V, Chetvernin V, Choi J, Cox E, Ermolaeva O, Farrell CM, Goldfarb T, Gupta T, Haft D, Hatcher E, Hlavina W, Joardar VS, Kodali VK, Li W, Maglott D, Masterson P, McGarvey KM, Murphy MR, O'Neill K, Pujar S, Rangwala SH, Rausch D, Riddick LD, Schoch C, Shkeda A, Storz SS, Sun H, Thibaud-Nissen F, Tolstoy I, Tully RE, Vatsan AR, Wallin C, Webb D, Wu W, Landrum MJ, Kimchi A, et al. 2016. Reference sequence (RefSeq) database at NCBI: current status, taxonomic expansion, and functional annotation. Nucleic Acids Res 44:D733–D745. doi:10.1093/nar/gkv1189. 26553804 PMC4702849

[40] Brettin T, Davis JJ, Disz T, Edwards RA, Gerdes S, Olsen GJ, Olson R, Overbeek R, Parrello B, Pusch GD, Shukla M, Thomason JA, III, Stevens R, Vonstein V, Wattam AR, Xia F. 2015. RASTtk: a modular and extensible implementation of the RAST algorithm for building custom annotation pipelines and annotating batches of genomes. Sci Rep 5:8365–8365. doi:10.1038/srep08365. 25666585 PMC4322359

[41] Camacho C, Coulouris G, Avagyan V, Ma N, Papadopoulos J, Bealer K, Madden TL. 2009. BLAST+: architecture and applications. BMC Bioinformatics 10:421. doi:10.1186/1471-2105-10-421. 20003500 PMC2803857

