# Supplementary figures and images for: PlasmidHostFinder: Prediction of Plasmid Hosts Using Random Forest
Source: mSystems. 2022 Apr 6;7(2):e01180-21. doi: 10.1128/msystems.01180-21 (PMC9040769; doi:10.1128/msystems.01180-21)

Tree scale: 100000

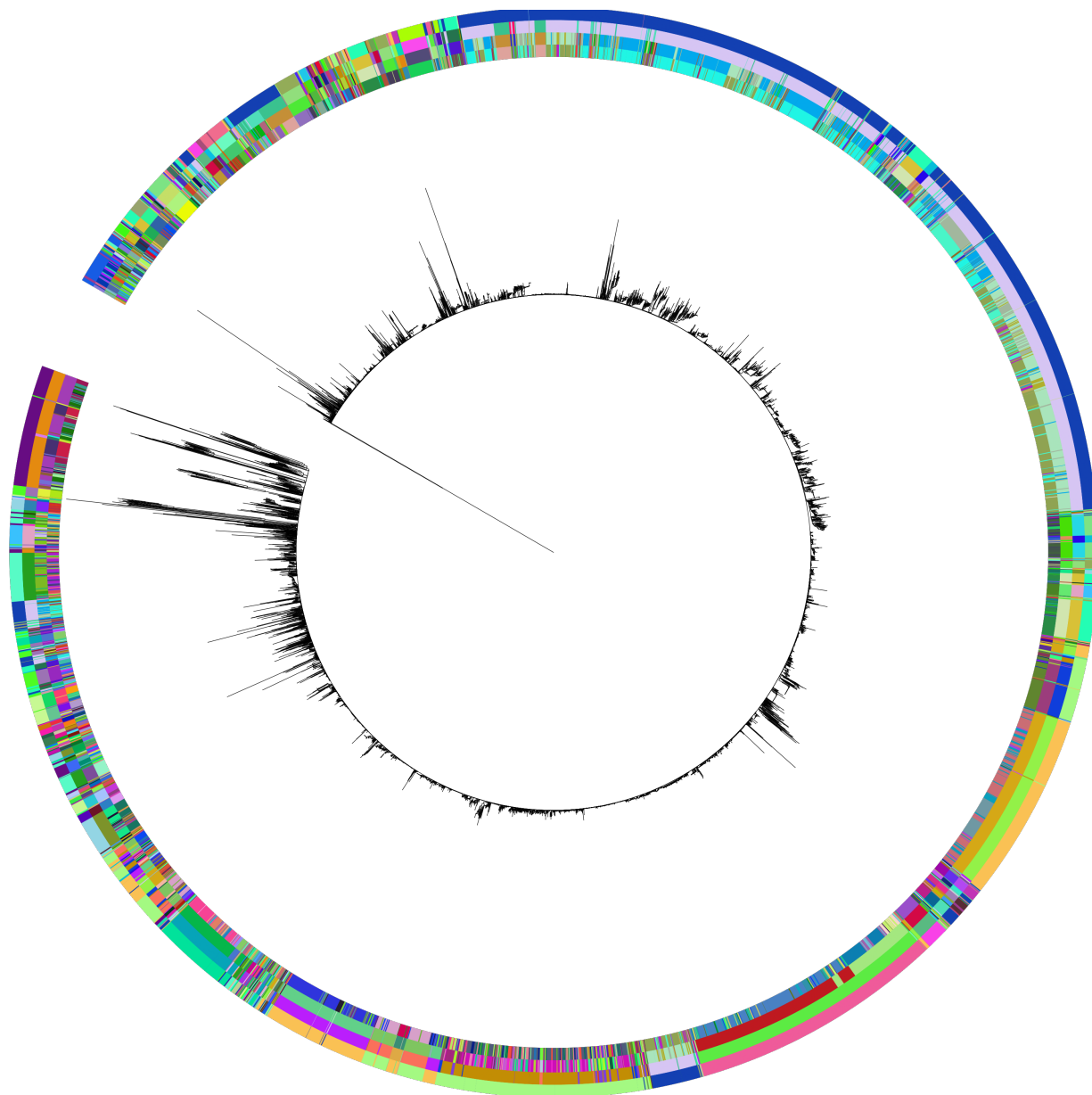

Supplement: FIG S1 [file msystems.01180-21-sf001.pdf]

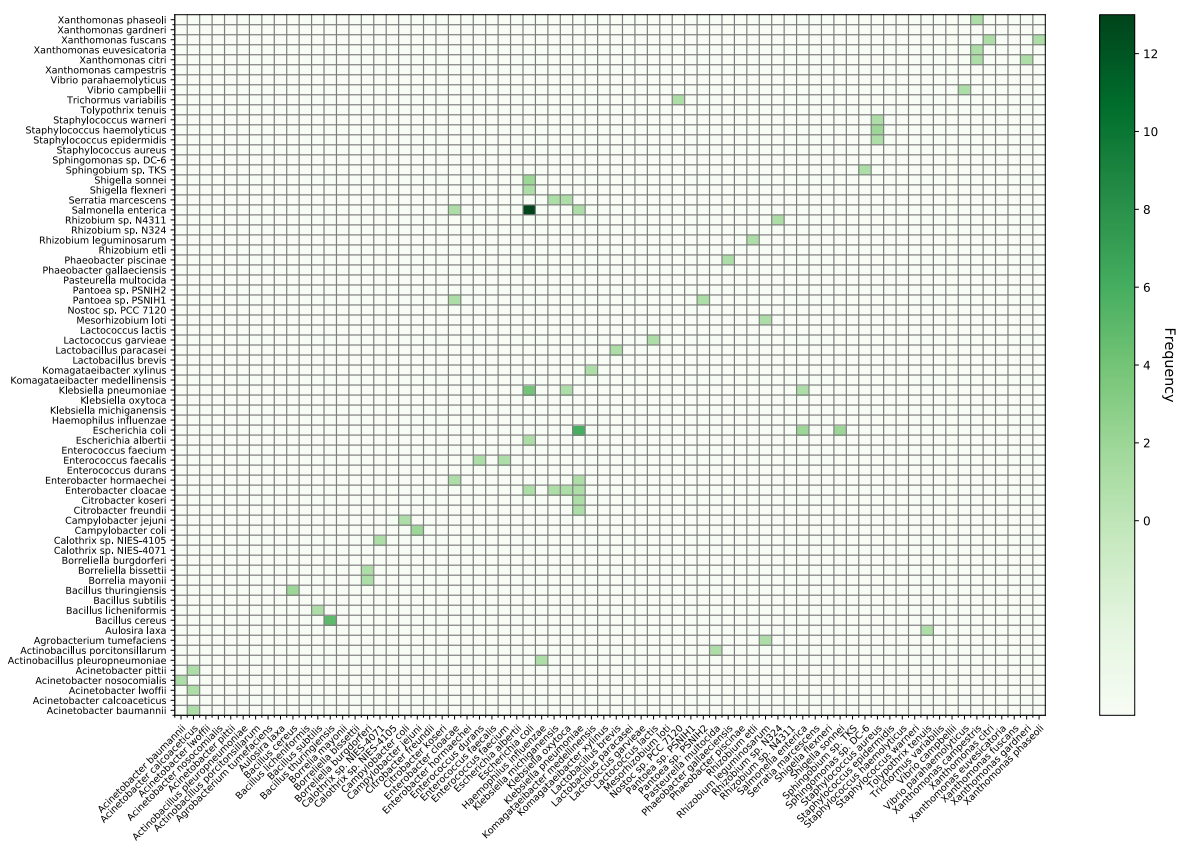

Supplement: FIG S3 [file msystems.01180-21-sf003.pdf]

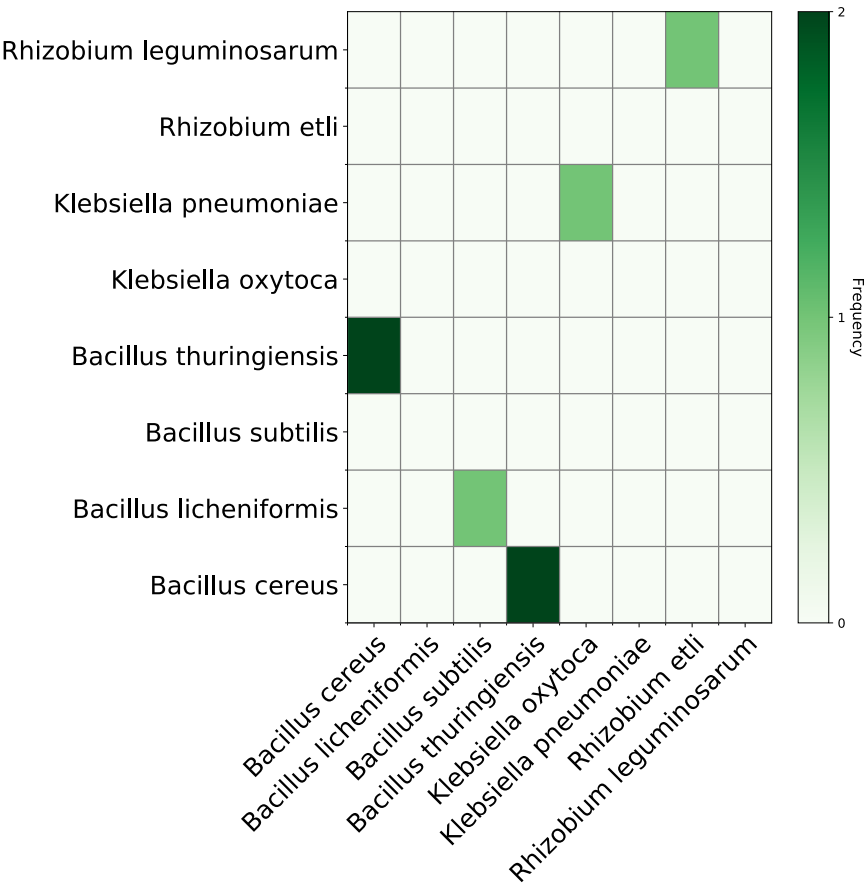

Supplement: FIG S4 [file msystems.01180-21-sf004.pdf]

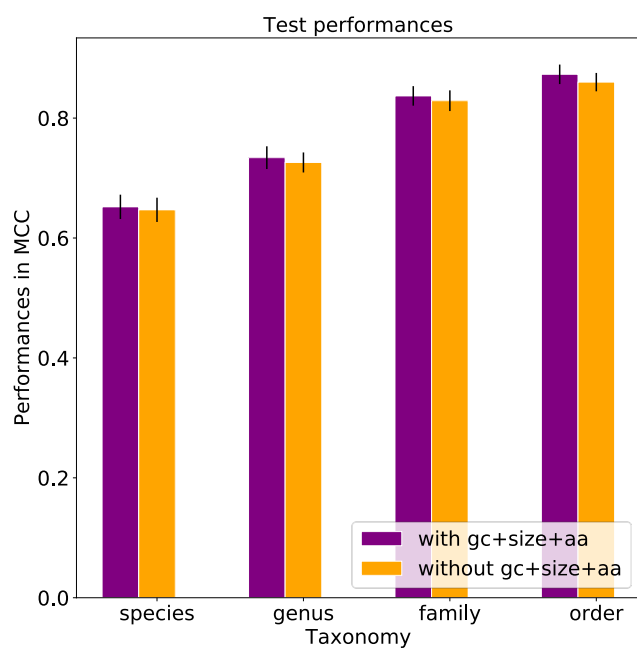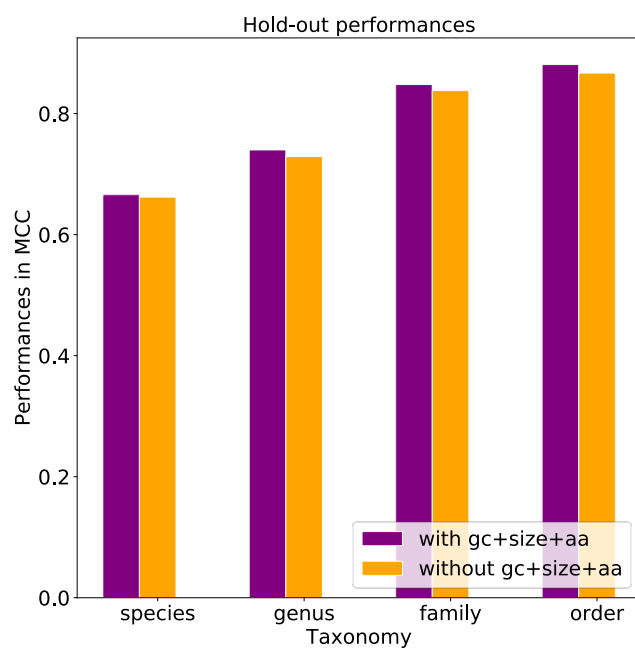

Supplement: FIG S2 [file msystems.01180-21-sf002.pdf]

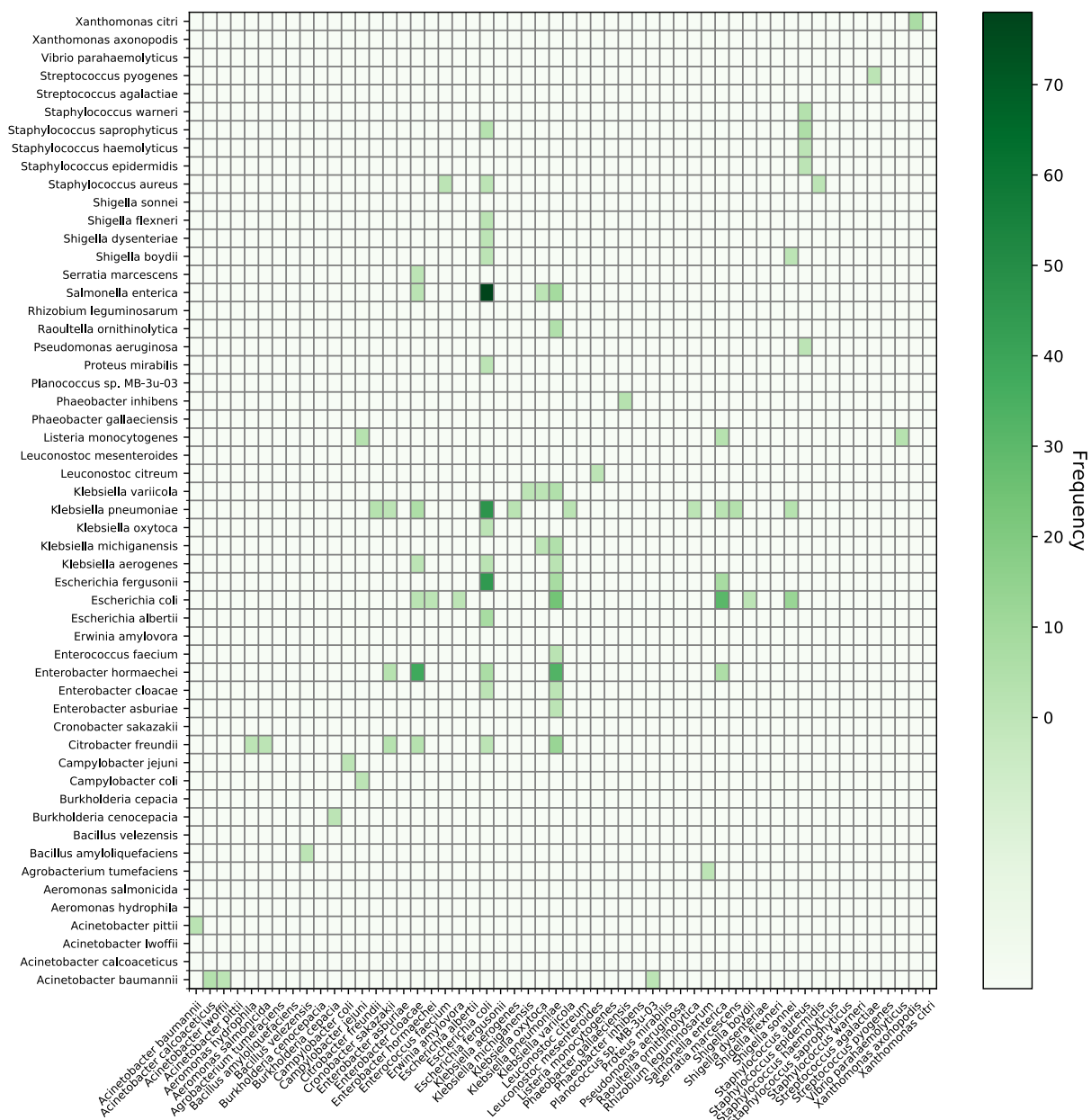

Supplement: FIG S5 [file msystems.01180-21-sf005.pdf]
